# Experience-Induced Remodeling of the Hippocampal Post-synaptic Proteome and Phosphoproteome

**DOI:** 10.1016/j.mcpro.2023.100661

**Published:** 2023-10-06

**Authors:** Seok Heo, Taewook Kang, Alexei M. Bygrave, Martin R. Larsen, Richard L. Huganir

**Affiliations:** 1 Solomon H. Snyder Department of Neuroscience, Johns Hopkins University School of Medicine, Baltimore, Maryland, USA; 2 Kavli Neuroscience Discovery Institute, Johns Hopkins University, Baltimore, Maryland, USA; 3 Department of Biochemistry and Molecular Biology, University of Southern Denmark, Odense, Denmark

**Keywords:** inhibitory avoidance, postsynaptic density, learning and memory, phosphoproteomics

## Abstract

The postsynaptic density (PSD) of excitatory synapses contains a highly organized protein network with thousands of proteins and is a key node in the regulation of synaptic plasticity. To gain new mechanistic insight into experience-induced changes in the PSD, we examined the global dynamics of the hippocampal PSD proteome and phosphoproteome in mice following four different types of experience. Mice were trained using an inhibitory avoidance (IA) task and hippocampal PSD fractions were isolated from individual mice to investigate molecular mechanisms underlying experience-dependent remodeling of synapses. We developed a new strategy to identify and quantify the relatively low level of site-specific phosphorylation of PSD proteome from the hippocampus, by using a modified iTRAQ-based TiSH protocol. In the PSD, we identified 3938 proteins and 2761 phosphoproteins in the sequential strategy covering a total of 4968 unique protein groups (at least two peptides including a unique peptide). On the phosphoproteins, we identified a total of 6188 unambiguous phosphosites (75%<site-localization probability). Strikingly, of the significantly IA-regulated phosphoproteins, a large fraction of these displayed an overall decrease in phosphorylation level. Bioinformatic analysis of proteins and phosphoproteins that were regulated by IA were annotated for involvement in the regulation of glutamate receptor functionality, RHO GTPase cycle, and synaptic plasticity. We also identified synaptic kinases, phosphatases, and their respective phosphosites regulated by IA training or immediate shock. Furthermore, we found that AMPA receptor surface expression was regulated by Mg2+/Mn2+ dependent protein phosphatase 1H (Ppm1h). Together, these results unravel the dynamic remodeling of the PSD upon IA learning or immediate shock and serve as a resource for elucidating the synaptic proteome dynamics induced by experience-dependent plasticity.

## Highlights

• Proteome and phosphoproteome of mouse hippocampal PSD following IA training and non-associative immediate shock.
• 3938 proteins and 2761 phosphoproteins were identified and quantified in the hippocampal PSD fractions.
• Kinases, phosphatases, and their phosphorylation status were dynamically and significantly regulated by IA and immediate shock.
• Functional validation shows that the protein phosphatase *Ppm1h* is linked to the regulation of synaptic plasticity *in vitro* and *in vivo.*

## Graphical Abstract

**Figure:**
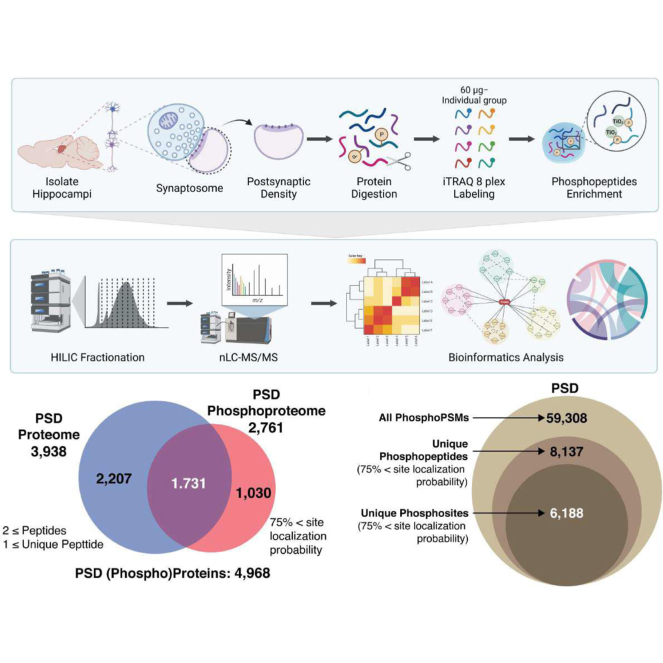

## In Brief

Heo et al. characterized the remodeling of postsynaptic proteome and phosphoproteome using quantitative proteomics and phosphoproteomics combined with subcellular protein fractionation and bioinformatics analysis and identify a highly dynamic regulation of synaptic protein phosphorylation at the postsynaptic density following IA training and immediate shock.

The dynamic tuning of synaptic strength, through processes known as synaptic plasticity, is crucial for learning and memory (1). Long-term potentiation (LTP) and long-term depression (LTD) are the two most studied forms of synaptic plasticity, arising from dynamic changes in neurons, including gene expression, protein trafficking, and post-translational modifications (PTMs) (2, 3). The postsynaptic density (PSD) is an essential structure of excitatory synapses which is composed of both membrane and sub-membranous components. Biochemical and molecular biological studies have identified a number of proteins in the PSD, including neurotransmitter receptors, scaffold proteins, cytoskeleton proteins, and signaling molecules, which together regulate synapse function, *i.e.*, the communication between the pre- and post-synapses. Biochemical enrichment of PSD fractions and advances in proteomic approaches contribute to the expansion of our understanding of the synaptic proteins enriched in the PSD and their PTMs (4). However, the dynamics of PTM in the PSD upon various behavioral tasks in the brain, such as experience-dependent activity changes are poorly understood, and could potentially shed light on important signaling pathways for learning and memory processes in the brain.

LTP at the Schaffer collateral pathway between CA3 and CA1 pyramidal neurons in the hippocampus is the best-characterized form of synaptic plasticity to date, both *in vitro* and *in vivo*. For example, LTP at CA3-CA1 synapses has been observed *in vivo* following inhibitory avoidance (IA) training in rats (5). The emotionally motivated learning following single-trial IA training is hippocampus-dependent (6) and robust and long-lasting (7). Memories formed by IA training are dependent on both protein synthesis and degradation. For example, studies have shown that inhibition of protein synthesis with protein synthesis inhibitors (*e.g.*, anisomycin) infused in the hippocampus or amygdala impaired consolidation, re-consolidation, and extinction of IA-memories (8, 9, 10). In addition, proteasome-mediated protein degradation is also required for intact IA-memory (11, 12). Protein phosphorylation/dephosphorylation of synaptic proteins also plays an important role in regulating the strength of synaptic connections (13). The function of reversible protein phosphorylation mediated by kinases and phosphatases has been studied for decades and it is clear that phosphorylation is critically important for learning and memory (14, 15). It is known that phosphorylation of different synaptic proteins is involved in different processes during memory formation (14, 16). These data suggest that IA-learning and subsequent memory formation require both synthesis and degradation of proteins, coupled with proper regulation of synaptic protein by PTMs such as phosphorylation.

Previous studies in mice have shown that IA induces changes in gene expression of c-Fos, Arc, Homer1a, Na^+^/K^+^-ATPase subunits, and glucose transporter type 1 (17, 18). In rats (5, 19) and mice (20), IA training leads to the recruitment of AMPA-type glutamate receptors (AMPARs) to the synaptosomal plasma membrane fraction. In addition, GluA1 phosphorylation such as at the CaMKII site Ser831 is elevated following IA training (5, 19). IA training also increased hippocampal CaMKII activity in the early phase of memory formation (19). These findings suggest that various synaptic proteins and their phosphorylation states are involved in IA-mediated learning and memory formation. However, our knowledge of synaptic proteins and their phosphorylation is limited to only a few synaptic proteins which are extensively studied.

Recent technological advances in mass spectrometry-based proteomics, including the development of high-resolution mass spectrometry (HR-MS) instruments and tools for quantitative assessment of protein phosphorylation (21), alongside improvements in bioinformatics, enable unbiased characterization of proteins and their phosphorylation in the brain with unprecedented depth (22, 23, 24, 25). The power of proteomic approaches is being harnessed to identify how synaptic proteins and their phosphorylation change with learning when combined with behavioral testing and pharmacological manipulations (26, 27, 28, 29, 30, 31, 32).

Here we used HR-MS combined with isobaric tags for relative and absolute quantification (iTRAQ) and TiO_2_-based phosphopeptide enrichment (33, 34, 35). This approach enables to probe of PSD-specific proteomic and phosphoproteomic remodeling following IA training through tandem MS scans that result in identifying the PSD peptides and phosphopeptides in the fear-conditioning mouse hippocampus. We identified a subset of significantly regulated PSD proteins and phosphoproteins which showed decreased abundance 1 h after IA training. Pathway analysis highlighted significantly enriched cellular functions related to synaptic plasticity, such as regulation of neurotransmitter receptor and ion transporter activity. Further analysis identified the involvement of distinct kinases and phosphatases (*e.g.*, Ppm1h), along with their phosphorylation sites, for the early phase of memory formation. This resource provides a novel perspective on IA- or immediate shock-associated hippocampal PSD proteome and phosphoproteome dynamics, revealing that large fractions of the synaptic proteins are differentially affected by different types of experiences.

## Experimental Procedures

### Experimental Design and Statistical Rationale

**Fig. 1:**
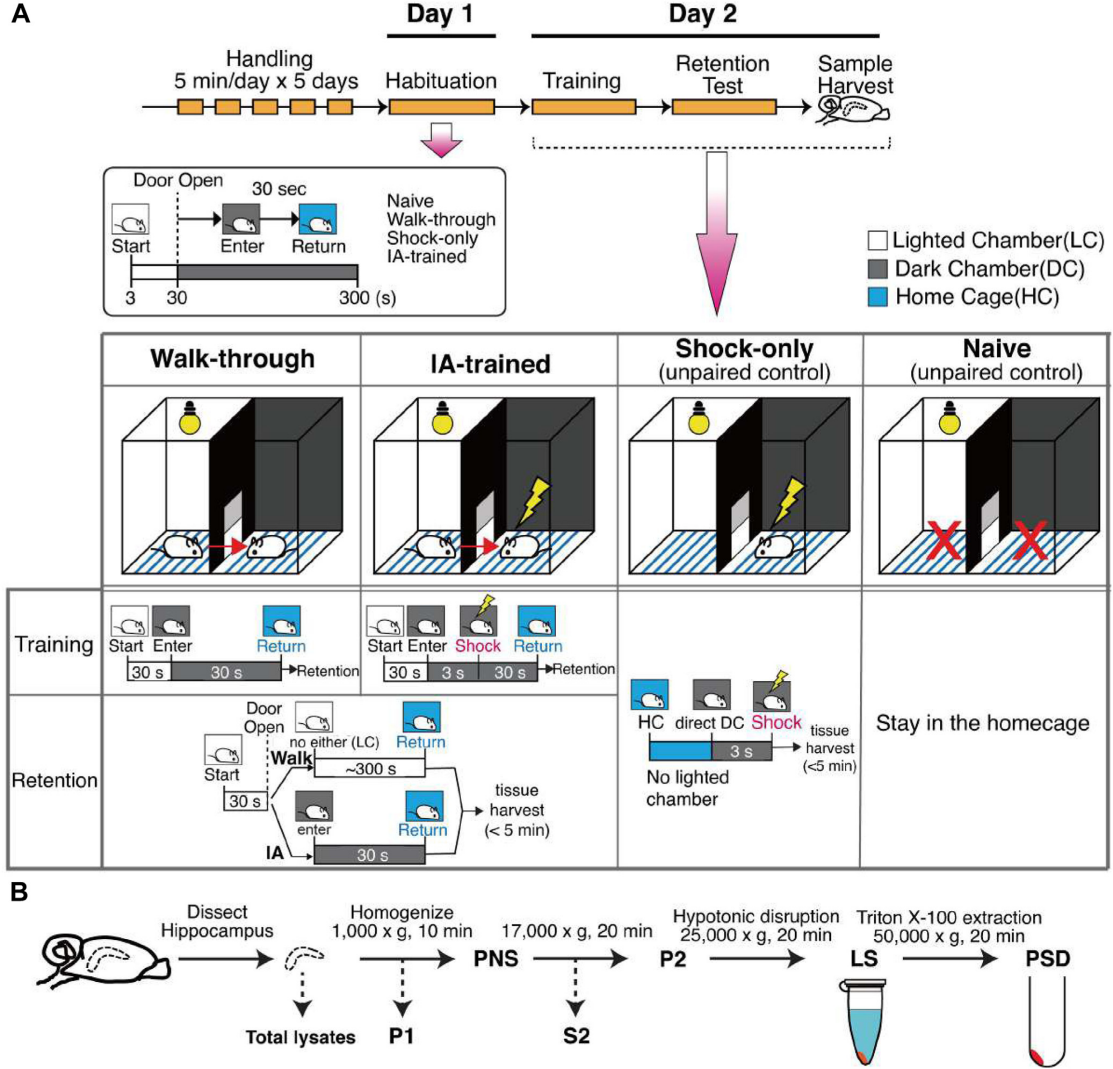

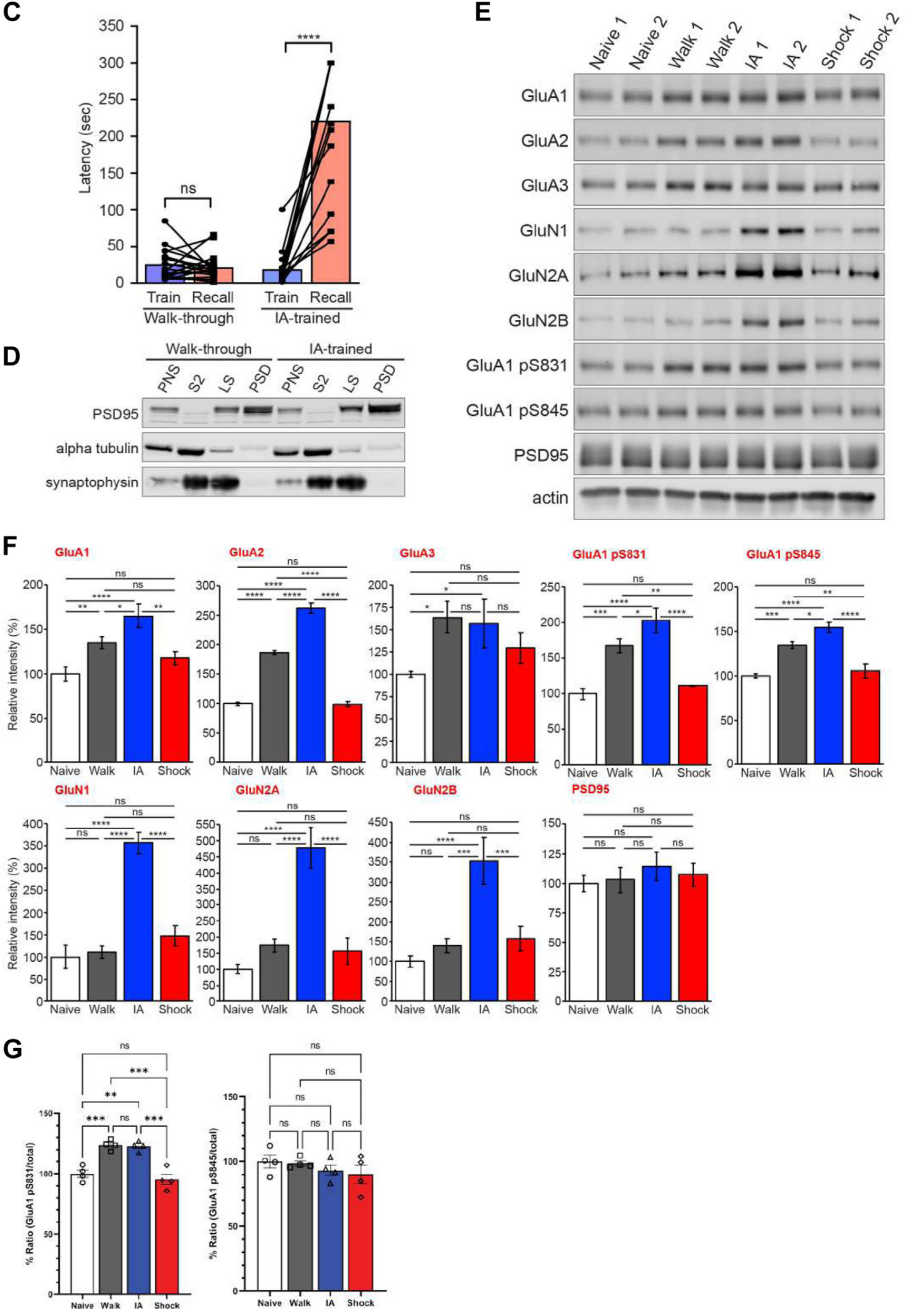
**Experience-dependent dynamics of synaptic proteins**. *A*, schematic of IA task. The behavioral test is composed of three sessions. Session #1 is handling, Session #2 is habituation, Session #3 is training and retention test followed by sample harvest. Mice were separated into four groups (Walk-through, IA-trained, Shock-only, Naïve; n = 7/group, three mice for proteomic analysis, four mice for Western Blot analysis) (see Experimental Procedures for details). *B*, schematic of subcellular fractionation for PSD preparation. (P1: nuclear fraction, PNS: post-nuclear supernatant, S2, cytosol, P2: membrane/crude synaptosome, LS: lysed synaptosome, PSD: postsynaptic density). *C*, behavioral results of IA training. The bar graph shows the latency for mice to cross to the dark side of the chamber, a measure of IA memory formation. The walk group (*left*) showed no significant changes in latency between the train (*blue*) and recall test (*red*). IA group (*right*) showed a significant increase in latency after training (n = 9/group; adjusted *p*-value, ∗∗∗∗*p* < 0.001 by 2-way ANOVA). *D*, validation of the PSD fraction quality in representative mice from Walk (*left*) and IA (*right*) groups. Note the enrichment of PSD-95 and the exclusion of alpha-tubulin in the PSD fraction. *E* and *F*, representative Western blots quantification of key synaptic proteins and their phosphorylation status in the PSD fraction. Western blot analysis to show changes of AMPAR, NMDAR and phospho-AMPAR. Error bars display ± SEM of four biological replicates (n = 4; adjusted *p*-value, ∗*p* ≤ 0.05, ∗∗*p* ≤ 0.01, ∗∗∗*p* ≤ 0.005, and ∗∗∗∗*p* ≤ 0.001 by 1-way ANOVA). *G*, ratio of phosphorylated GluA1 normalized to total GluA1 in the PSD. Error bars display ±SEM of four biological replicates (n = 4; adjusted *p*-value, ∗*p* ≤ 0.05, ∗∗*p* ≤ 0.01, ∗∗∗*p* ≤ 0.005, and ∗∗∗∗*p* ≤ 0.001 by 1-way ANOVA).

All experiments were approved by the local Ethics Committee in accordance with Danish legislation. Animal care, use, and experimental protocols were approved by the Institutional Animal and Use Committee (IACUC) of Johns Hopkins University. See Figure 1*A* for a summary of IA experiments. In this study, we used four experimental groups: IA-trained animals (IA), a walk-through group that received no shock when crossing from the light to the dark (Walk), unpaired control (Shock-only), and untrained mouse (Naïve) groups. Four biological replicates (Western blot) and three biological replicates (proteomics and phosphoproteomics) were performed per animal condition and experimental analysis. For proteomics and phosphoproteomics analysis, each biological replicate consisted of 60 μg of proteins extracted from the PSD fraction of mouse hippocampus from the four experimental groups (Naïve, Walk, Shock-only, and IA). Our proteomics and phosphoproteomics analyses were comprehensive and carried out with sufficient replicates as in our previous isobaric labeling-based studies (33, 34, 35). To further refine the criteria for statistical significance of protein expression and phosphopeptides fold changes, significantly regulated proteins or phosphopeptides were accepted if the z-test for adjusted *p*-value was <0.05 (95% confidence) with the Benjamini and Hochberg correction in a normal distribution. All statistical tests for the analyses of biochemical and behavioral data were performed using the Prism nine software package (version 9.4.1; GraphPad). For Western blotting analysis, one-way ANOVAs were used for the analyses of glutamate receptor isoform levels in IA experiments and Ppm1h levels in different subcellular fractions in the chemical LTP experiment. Tukey post-hoc tests were used to analyze significant changes in target protein levels. Two-way ANOVAs were used to compare the behavioral differences in mice, and post-hoc Tukey tests were used to identify significant changes in latency in IA between control and trained groups. Student t-tests were employed to analyze (1) the effect of Ppm1h overexpression in surface expression of glutamate receptor isoforms, (2) the effect of contextual fear conditioning (cFC) on freezing behavior, and (3) the effect of enriched environment (EE) or cFC on the subcellular distribution of Ppm1h.

### Animal Use

For inhibitory avoidance experiments, C57BL/6 mice (purchased from Charles River Laboratories) were delivered at age 8 weeks and group-housed for 2 weeks until IA testing. Timed pregnant Sprague Dawley rats (purchased from Envigo, formerly Harlan Laboratories) were used for primary neuronal cultures at embryonic day 18 (E18) as described below. All animals were group-housed in a standard 12 h light/12 h dark cycle. IA testing was conducted during the dark phase.

### Inhibitory Avoidance (IA)

Schematics of the IA protocol are shown in Figure 1*A*. Mice were handled for 5 min on each of the five consecutive days before beginning experiments. The inhibitory avoidance (IA) testing cage consisted of a rectangular chamber (35.56 cm wide × 17.78 cm deep × 30.48 cm high, Passive avoidance cage for mouse from Coulbourn Instruments; 24.13 cm wide × 20.32 cm deep × 20.32 cm high, GEMINI Avoidance system from San Diego Instruments) divided into two separate compartments, “light” and “dark” compartments. The light compartment was built with transparent Plexiglas and illuminated with a bright overhead stimulus light, while the dark compartment was built with nontransparent Plexiglas and was not illuminated. The compartments were separated by a guillotine door, and both compartments were equipped with metal grid floors connected to an electric generator source that delivered an electric shock (1 mA, 2 s). IA testing cage was controlled by Graphic State 2, a state notation program (Coulbourn Instruments). For our experiment, we adopted three-step IA protocol that consisted of three individual sessions, habituation, acquisition, and retention (recall). The latency to enter the dark compartment was recorded as an index of memory consolidation using ANY-maze behavior tracking software.

For habituation (day 1), a mouse was placed in the light side of the chamber facing the wall of the opposite side of the guillotine door. After 30 s the door was opened and the mouse was allowed to explore until it entered the dark compartment. The door closed immediately after the mouse entered the dark side and the mouse was returned promptly to the home cage after entering the dark compartment of the testing cage. Mice in the Walk-through, IA-trained, and Shock-only groups were habituated to the IA testing cage, and mice from the Naïve group remained in the home cage until they were sacrificed for tissue harvesting.

For acquisition (day 2), the mouse again was placed in the light compartment of the testing cage facing the wall on the opposite side of the guillotine door. The door was opened after 30 s, and the latency to cross to the dark side following the door opening was recorded. The guillotine door closed immediately after the mouse entered the dark compartment, and 3 s later the mouse in the IA-trained group received a foot shock (2 s, 1 mA). The mouse remained in the dark chamber for 30 s following foot shock for recovery, then it was returned gently to the home cage. The mouse in the Walk-through group did not get a foot shock and remained in the dark for 30 s. Animals in the ‘Shock-only’ group were directly placed in the dark side of the IA testing cage and given the same strength of foot shock (2 s, 1 mA) and were immediately removed from the IA chamber for tissue harvest.

For the retention/memory test, 1 h after training the mouse was reintroduced to the light compartment of the testing cage facing the wall on the opposite side of the guillotine door. The door opened 30 s after the mouse was placed in the light compartment, and the latency to step through to the dark compartment was recorded as a measure of memory retention (compared with step through latency of the acquisition trial). The maximum latency was set at 5 min, after which mice were returned to the home cage. The hippocampus was dissected within 5 min after completion of the retention test or immediate shock delivery or staying in the home cage. Mice were anesthetized with isoflurane for 15 s followed immediately by cervical dislocation. Brains were removed and hippocampi were dissected in ice-cold dissection media and immediately frozen with liquid nitrogen. Samples were kept at −80 °C until subcellular fractionation for PSD preparation.

### Enriched Environment (EE)

For all experiments, C57BL/6 male mice were aged 8 to 10 weeks. Mice (n = 18/group) were first handled and habituated to minimize stress-induced changes. Mice were then either left in their home cage (control) or allowed to explore an EE which is composed of novel objects, tubes, and strings of beads suspended from the cage lid in a large cage for 8 h during the wake period and then transferred back to the home cage. This is a physiologically relevant condition which expected to drive neuronal activity and synaptic plasticity (36, 37). On the next day, mice were exposed to the EE cage with different sets of novel objects in a different arrangement for 8 h. This 2-day EE cycle was repeated for 2 weeks. On the last day, mice were exposed to the EE chamber and then anesthetized by inhalation of isoflurane for 15 s followed immediately by cervical dislocation. Brains were removed and hippocampi were dissected in ice-cold dissection media and immediately frozen with liquid nitrogen. Samples were kept at −80 °C until being subject to subcellular fractionation to isolate the PSD.

### Contextual Fear Conditioning

Fear conditioning was performed using Ugo Basile Fear Conditioning System (Stoelting Co) and recorded using ANY-maze behavior tracking software as previously described with slight modifications (38). Briefly, mice were handled for 5 min on each of the five consecutive days before beginning experiments (n = 4/group). For habituation, mice were placed in the cFC chamber and left them explore the chamber for 5 min. Mice were returned promptly to the home cage after 5 min habituation. For training, mice in the control group again were placed in the cFC chamber and left them explore the chamber for 5 min. Mice in the training group were placed in the cFC chamber for 2 min (baseline) and delivered a foot shock (2 s, 0.75 mA) five times. The interstimulus interval was 30 s. For the retention test, mice were reintroduced to the cFC chamber 1 h after training, but no foot shock was delivered during 5 min testing. The time that mice showed freezing behavior was measured as a measure of memory retention. The hippocampi were dissected 5 min after completion of the retention test and were kept at −80 °C until being subject to subcellular fractionation to isolate the PSD.

### Dissociated Rat Neuronal Culture

Cortical neurons obtained from pregnant wild-type Sprague Dawley rats (purchased from Envigo) at embryonic day 18 were initially prepared in Neurobasal media (Invitrogen) supplemented with 2% B-27, 2 mM GlutaMax, 50 U/ml penicillin, 50 mg/ml streptomycin, and 5% horse serum (Invitrogen) and plated onto poly-L-lysine-coated tissue culture dishes at a density of 800,000 cells per well. Cortical neurons were then transferred and maintained in a humidified tissue culture incubator at 37 °C in a 95% air and 5% CO_2_ mixture; 5 mM FDU (5-Fluoro-2′-Deoxyuridine and 5 mM Uridine; Sigma) was added at DIV4 to inhibit glia proliferation and cells were thereafter maintained in NM1 (Neurobasal media with 2% B-27, 2 mM GlutaMax, 50 U/ml penicillin, 50 mg/ml streptomycin, and 1% horse serum). Cultured cortical neurons were fed twice per week. Cortical neurons were grown for 18 to 19 days *in vitro* for induction of chemical LTP. For Ppm1h overexpression experiments, cortical neurons were electroporated with myc-Ppm1h construct at DIV0 using Rat Neuron Nucleofector kit (Lonza) following manufacturer’s manual, and cells were used when 2 to 3 weeks old.

For glycine-induced chemical LTP experiments, cortical neurons (DIV19–20) were first preincubated with Mg^2+^-ACSF (143 mM NaCl, 5 mM KCl, 10 mM HEPES [pH 7.42], 10 mM Glucose, 2 mM CaCl_2_, 1 mM MgCl_2_, 0.5 μM TTX, 1 μM Strychnine, and 20 μM BIC), followed by glycine treatment for 10 min (chemical LTP ACSF: 200 μM glycine/0 Mg^2+^), and returned to the original Mg^2+^-ACSF (0 glycine/1 mM MgCl_2_) for 30 min prior to surface biotinylation/lysis.

### Surface Biotinylation

Neurons were rinsed with ice-cold PBS containing 0.1 mM CaCl_2_ and 1 mM MgCl_2_ (pH 8.0) (PBS-CM), then incubated in PBS-CM containing 1 mg/ml Sulfo-NHS-SS-biotin (Thermo Fisher Scientific, 30 min, 4 °C). After the biotinylation reaction, neurons were rinsed with PBS-CM, and the biotinylation reaction was quenched in PBS-CM containing 50 mM glycine (2 x 5 min, 4 °C). Cells were lysed in RIPA buffer containing protease inhibitor cocktail (Roche), phosphatase inhibitor cocktail (Roche), and 1 μM okadaic acid, then cleared by centrifugation (17,000*g*, 10 min, 4 °C). Protein concentration of each lysate was quantified using BCA protein assay kit (Thermo Fisher Scientific), and equal amounts of protein were incubated overnight with NeutrAvidin-coupled agarose beads (Thermo Fisher Scientific) at 4 °C with gentle rotation. Beads were washed three times with ice-cold lysis buffer, and biotinylated proteins were eluted with 2× SDS sample buffer. Cell-surface or total proteins were then subjected to SDS-PAGE and analyzed by Western blot.

### Subcellular Fractionation and Western Blotting

For postsynaptic density preparation (Fig. 1*B*), hippocampi were dissected immediately following the memory retention test (one-hour following IA-training or walk-through) or immediate foot shock delivery (Shock-only group). The hippocampus from each mouse was homogenized individually using 20 strokes from syringes equipped with 26G × 3/8 (0.45 mm × 10 mm) needles in homogenization buffer (320 mM sucrose, 5 mM sodium pyrophosphate, 1 mM EDTA, 10 mM HEPES pH 7.4, 200 nM okadaic acid, 1 mM sodium orthovanadate, protease inhibitor cocktail (Roche), phosphatase inhibitor cocktail (Sigma-Aldrich)). The homogenate was then centrifuged at 1000*g* for 10 min at 4 °C to yield P1 (nuclear fraction) and post-nuclear supernatant (PNS) fractions. PNS fraction was further centrifuged at 17,000*g* for 20 min at 4 °C to yield P2 (membrane/crude synaptosome) and S2 (cytosol) fractions. P2 was resuspended in hypotonic resuspension buffer (Milli-Q water with 5 mM sodium pyrophosphate, 1 mM EDTA, 10 mM HEPES pH 7.4, 200 nM okadaic acid, 1 mM sodium orthovanadate, protease inhibitor cocktail (Roche), phosphatase inhibitor cocktail Roche)), then centrifuged at 25,000*g* for 20 min at 4 °C to yield lysed synaptosome (LS) fractions. Collected LS fractions were resuspended in resuspension buffer (50 mM HEPES pH 7.4, 5 mM sodium pyrophosphate, 1 mM EDTA, 200 nM okadaic acid, 1 mM sodium orthovanadate, protease inhibitor cocktail (Roche), phosphatase inhibitor cocktail (Roche)) and then mixed with an equal part of 1% Triton X-100 (containing protease and phosphatase inhibitors). This mixture was incubated at 4 °C with rotation for 10 min followed by centrifugation at 50, 000*g* for 20 min at 4 °C to yield PSD preparation. The final PSD pellet was resuspended in 50 mM HEPES pH 7.4 (containing protease and phosphatase inhibitors). The protein concentration from PSD fractions was determined using a BCA protein assay followed by biochemical analysis.

For Western blotting analysis, samples were quantified using BCA protein assay kit and loaded onto nine or 12% SDS-PAGE (depending on the molecular weights of the protein of interest). Proteins were transferred to the PVDF membrane, and the membranes were blocked with Odyssey blocking buffer for fluorescent detection for 1 h at room temperature. Primary antibodies were resuspended in Odyssey blocker/TBS-T (1X TBS supplemented with 0.2% Tween 20) mixture (Odyssey blocker: TBS-T = 1 : 1) and incubated overnight at 4 °C with gentle rocking. Primary antibodies were removed and membranes were washed followed by IRDye-conjugated secondary antibody incubation in blocking solutions. For primary antibodies where IRDye-conjugated secondary antibodies were not available, membranes were first probed with HRP (horseradish peroxidase)-conjugated secondary antibodies followed by re-probing with IRDye-conjugated anti-HRP antibody. Blots were developed using either LI-COR Odyssey CLx Imaging system (LI-COR).

### Sample Preparation for Mass Spectrometry Analysis

#### In-Solution Trypsin and Lys-C Digestion

PSD fractions isolated from mouse hippocampi were lysed, reduced, and predigested in 6 M Urea, 2 M Thiourea, containing 10 mM Dithiothreitol and 2 μl Lys-C endopeptidase supplemented with PhosSTOP phosphatase inhibitor for 2 h at room temperature (RT). Thereafter, the lysates were diluted 10 times using 20 mM Triethylammonium bicarbonate buffer (TEAB; pH adjusted to 7.5) and tip-sonicated for 2 x 20 s on ice. Samples were then alkylated by 20 mM iodoacetamide for 20 min in the dark before digestion with 2% (w/w) trypsin overnight at 37 °C.

#### iTRAQ Labeling of Peptides

The peptide concentration was measured by Qubit Fluorometric protein assay according to the manufacturer’s instructions. A total of 60 μg was aliquoted from all samples (4 groups from total lysates and PSD fractions) and lyophilized before labeling with iTRAQ eight plex kit (AB Sciex). Three biological replicates were made and labeling was performed as follows: total naïve 113, total walk-through 114, total shock-only 115, total IA-trained 116, PSD naïve 117, PSD walk-through 118, PSD shock-only 119, and PSD IA-trained 121. The labeling was performed according to the manufacturer’s protocol, and complete labeling was validated by running combined aliquots on MALDI MS (Bruker Daltonics, Germany). An equal amount (60 μg) of protein per sample was mixed in equal ratios and stored at −20 °C until phosphopeptide enrichment.

#### Enrichment of Phosphorylated Peptides

The purification of phosphopeptides was performed according to a slightly modified TiSH (TiO_2_-SIMAC-HILIC) phosphopeptide enrichment procedures (21, 33, 35, 39), in which nonmodified peptides are first separated from phosphopeptide species using TiO_2_ beads. Briefly, the lyophilized iTRAQ labeled sample was made up to 1 ml loading buffer [1 M glycolic acid, 80% acetonitrile (ACN), 5% TFA] and added with TiO_2_ beads at 0.6 mg/100 μg (bead/peptide), and incubated at RT for 10 min. The suspension was centrifuged for 15 s in a table centrifuge and the supernatant was loaded onto a second batch of TiO_2_ (containing half the amount of TiO_2_ as initially used) and incubated at RT for 15 min. The two batches of TiO_2_ were washed with 100 μl of washing buffer 1 [80% ACN, 1% trifluoroacetic acid (TFA)] and centrifuged for 15 s in a tabletop centrifuge. The supernatant was removed, and the beads were washed with 100 μl washing buffer 2 (10% ACN, 0.1% TFA) and centrifuged for 15 s in a tabletop centrifuge. The supernatant was removed, and the beads were dried in a vacuum centrifuge for 5 min. The bound peptides were eluted with 100 μl of 1% ammonium hydroxide for 15 min and then centrifuged at 1000 g for 1 min. The eluted peptides were passed over a C8 stage tip (40) to retain the TiO_2_ beads and dried by vacuum centrifugation to produce the enriched phosphopeptide fraction. The flow through from the initial loading buffer (containing nonmodified peptides) and washes were combined and dried by vacuum centrifugation to produce the nonmodified peptide fraction. The nonmodified peptide fraction was acidified with TFA and desalted on an R3 stage tip column before HILIC fractionation.

#### Sample Desalting

Samples were desalted before HILIC fractionation. The desalting columns were self-made by inserting a small plug of C18 material into the constricted end of a 200 μl tip and packed with a mixture of R2 and R3 reversed-phase resin applying manual air pressure with a syringe, followed by an optimized desalting procedure (39). Briefly, the samples were acidified before loading onto the columns (equilibrated with 0.1% TFA), followed by washing with 0.1% TFA, and peptides were eluted using 60% ACN and 0.1% TFA and were lyophilized before further processing.

#### Hydrophilic Interaction Liquid Chromatography

The phosphorylated and the nonmodified peptide samples were subjected to fractionation using hydrophilic interaction liquid chromatography (HILIC) (33). Briefly, these samples were resuspended in 90% ACN, 0.1% TFA (Solvent B) and loaded onto a 450 μM OD × 320 μM ID × 17 cm micro-capillary column packed with TSKgel Amide-80 resin material using an Agilent 1200 Series HPLC. Peptides were separated using a gradient from 100 to 60% Solvent B (Solvent A: 0.1% TFA) running for 30 min at a flow-rate of 6 μl/min. The fractions were automatically collected in a 96 well plate at one-minute intervals after UV detection at 210 nm.

### LC-MS/MS, Proteomic Data Handling and Bioinformatic Analysis

#### Reverse-phase nanoLC-ESI-MS/MS Analysis

All fractions were redissolved in buffer A (0.1% FA) and analyzed using an nLC-MS/MS system consisting of an Easy-nLC and an Orbitrap Fusion Lumos (phospho-proteome) or a Q-exactive HF (proteome) mass spectrometers (MS) were used separately to increase the speed of analysis. The samples were loaded onto a 2 cm pre-column (100 μm inner diameter) and separated on a 17 cm fused silica capillary column (75 μm inner diameter). All columns were homemade and packed with ReproSil-Pur C18 AQ 3 μm reversed-phase resin material. The peptides were eluted using 73 to 133 min gradients from 1 to 40% buffer B (95% ACN, 0.1% FA) and introduced into the MS instrument *via* nanoelectrospray according to the intensity of each HILIC peptide fraction. A full MS scan in the mass area of 400 to 1400 Da was performed in the Orbitrap with a resolution of 120,000, an AGC target value of 5 × 10^5^, and a maximum injection time of 100 ms. For each full scan, “Top speed” mode was selected for higher energy collision dissociation (HCD). The settings for the HCD were as follows: AGC target value of 3 × 10^4^, maximum injection time of 60 ms, isolation window of 1.2 Da, and normalized collision energy of 38. All raw data were viewed in Xcalibur v4.0.

#### MS Data Processing and Statistical Analysis

The raw MS data sets were processed for protein/peptide identification using the Proteome Discoverer (PD, v. 3.0) and the Sequest HT algorithm with a peptide mass tolerance of 10 ppm, a fragment ion mass tolerance of 0.02 Da, and a false discovery rate (FDR) of 1% for proteins and peptides. All peak lists were searched against the UniProtKB/Swiss-Prot database of mouse sequences (04/2023, UP000000589, Gene count 21,949, Protein count 55,260) with decoy using the parameters as follows: enzyme, trypsin; maximum missed cleavages, two; fixed modification, carbamidomethylation (C), iTRAQ tags (K, peptide N termini); variable modifications, oxidation (M) and phosphorylation (S, T, Y). Data sets with raw MS values were filtered to remove potential errors using several criteria. For relative protein quantification, the output Excel sheet file was exported from PD, and then filtered as follows: each unique protein group contains at least two peptides and one unique peptide and phosphosite localization probability with high confidence (at least 75%) using ptmRS node in PD. Protein relative expression values from the respective unique peptides (only in a single protein) were calculated by summing all peptide intensities of each protein and normalized to the number of the total intensity of each group to estimate the relative amounts of the different proteins within the sample. The resulting ratios were log-transformed (base = 2) to achieve a normal distribution, and then log_2_ ratios were averaged per unique protein, phosphopeptide, or site-specific phosphorylation for subsequent analysis. Three biological replicates were performed. All differentially expressed proteins and altered phosphopeptides were defined using statistical methodology (z-test for adjusted *p*-value <0.05 with the Benjamini-Hochberg correction).

#### Bioinformatic Processing and Data Analysis

Gene Ontology (GO) Cellular Component annotation enrichment analysis was performed using the SynGO and UniProt databases. DAVID GO analysis including the Reactome Pathway Database was used to functionally annotate genes implicated in biological functions, using an FDR threshold of 0.05. The regulated proteins and phosphorylation were searched against the STRING database (version 11.5) and BioGRID database for protein-protein interactions and upstream molecule analysis.

We used the FASTA sequences of the kinase domains retrieved from the KinBase resource or phosphatase domains from the Unitprot database and aligned them by ClustalX2.1 using default parameters for multiple alignment and bootstrapping N-J tree. Kinase or phosphatase sequences were visualized by phylogenetic distances using the Interactive Tree of Life (ITOL) tool (https://itol.embl.de).

## Results

### Inhibitory Avoidance Training Induces Changes in Glutamate Receptors and Their Phosphorylation Status

Initially, we sought to confirm that IA training induced a robust memory 1 h after training and that we could replicate changes in synaptic proteins that have been reported previously (5, 17). The IA training paradigm consisted of three sessions: (1) pre-testing habituation (5 min/day × 5 days), (2) training (IA, Walk, Shock groups), and (3) memory retention (recall) test. We used four experimental groups: IA-trained animals (IA), a walk-through group that received no shock when crossing from the light to the dark (Walk), shock only (Shock) and naïve group (Naïve) animals (Fig. 1*A*). One hour following training, IA memory was assessed by measuring the latency of mice to cross into the dark side of the chamber, after which mice were immediately euthanized and their hippocampi were harvested for PSD preparation (Fig. 1*B*). As expected, during the training session, mice from both Walk and IA groups showed short latencies to cross to the dark chamber, indicating a preference for a dark environment (photophobia). In contrast, during the memory recall session, the IA group showed significantly longer latencies compared to the Walk group (Fig. 1*C*). This demonstrates the robust one-trial learning induced by IA training.

Hippocampi harvested after the recall test was homogenized to prepare PSD fractions. Crude synaptosomes obtained from the PNS fraction were disrupted by hypotonic solution followed by PSD extraction using Triton X-100 (see Experimental Procedures; Fig. 1*B*). The quality of the PSD fraction was monitored by visualizing the enrichment of PSD-95 and depletion of α-tubulin and synaptophysin in PSD fractions compared to other intermediate fractions (Fig. 1*D*). Numerous studies have demonstrated that trafficking of different types of glutamate receptors contributes to LTP and other types of synaptic plasticity induced by learning (41, 42, 43, 44, 45, 46, 47, 48). We probed for changes in AMPA and NMDA receptors, and their phosphorylation status in hippocampal PSD fractions from control (Naïve, Walk and Shock) and IA-trained mice. We found a significant increase of GluA1, GluA2, and GluA3 following IA training compared to the naïve control group. Subunits of NMDA receptors, GluN1, GluN2A, and GluN2B also increased in the PSD following IA training. The well-characterized phosphorylation sites of GluA1 at Ser831 (pS831) and Ser845 (pS845) increased compared to all control groups (Fig. 1, *E* and *F*). The ratio of pS831 over total GluA1 increased in the Walk and IA group but not in the Shock group. In contrast, the ratio of pS845 over total GluA1 was not significantly different across all groups (Fig. 1*G*). This indicates that the phosphorylation of GluA1 in the PSD is differentially regulated depending on the phosphorylation site and that the increase in p845 is being driven by increased levels of total GluA1. All AMPA and NMDA receptor subunits that we tested showed a robust increase in PSD following IA training. Interestingly, GluA1, GluA2, GluA3, pS831, and pS845 of GluA1 also increased in the Walk group compared to the naïve and Shock group (Fig. 1, *E* and *F*). In contrast, no changes in the level of the synaptic scaffolding protein PSD-95 levels were detected. This validation indicates that IA training increases the targeting and phosphorylation of AMPA and NMDA receptors to the PSD, presumably underlying the expression of LTP *in vivo*.

### Quantitative Analysis of the PSD Proteome and Phosphoproteome

**Fig. 2:**
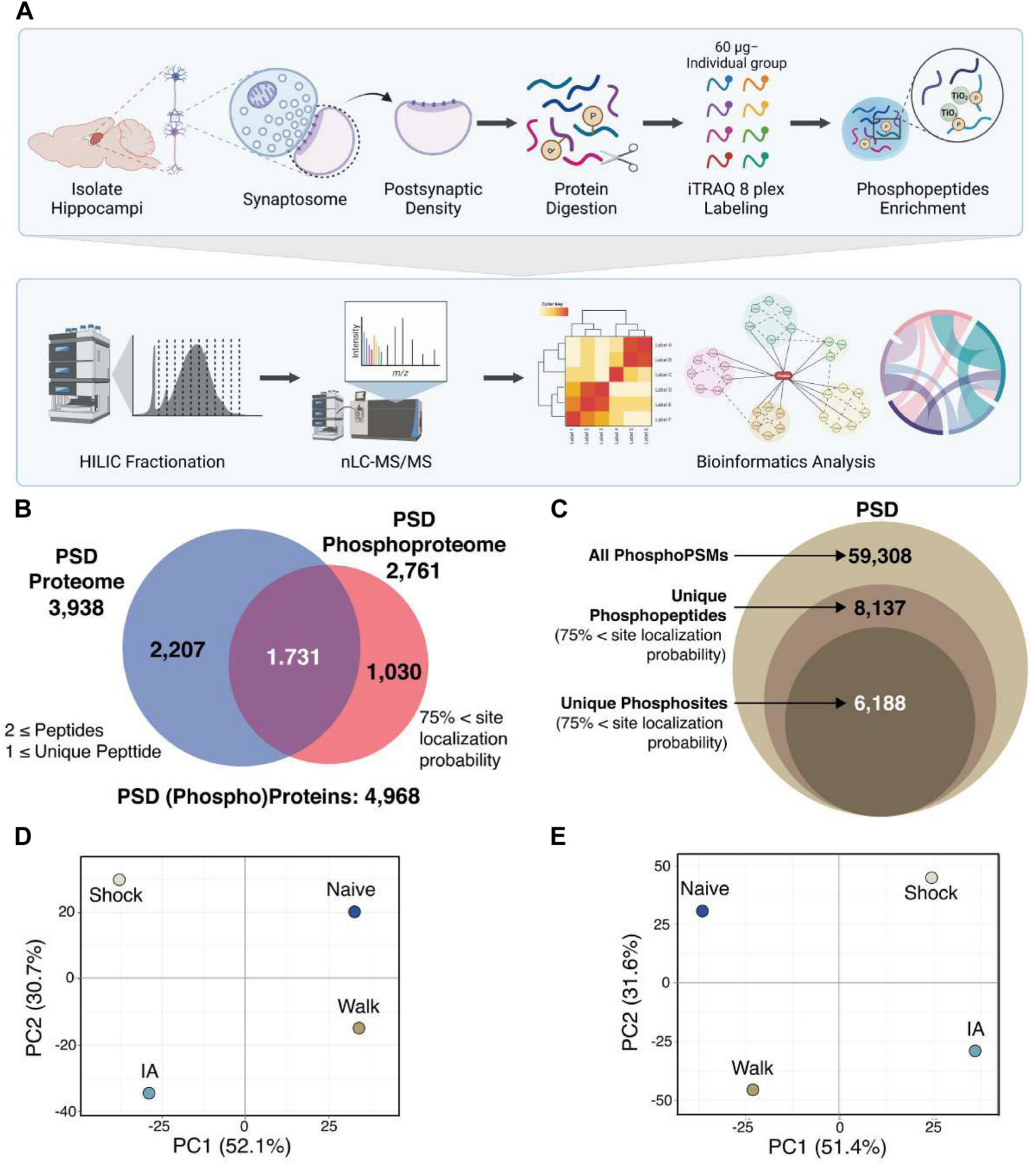
**Identification and quantification of experience-dependent proteome and phosphoproteome dynamics in hippocampal PSD fractions**. *A*, workflow of sample preparation and mass spectrometry (MS)-based phosphoproteomics analysis. PSD fractions were prepared from hippocampi dissected from individual mice from all four groups. Proteins were extracted and digested with trypsin/LysC to generate peptides for iTRAQ labeling. Multiplex-labeled peptide mixture was subjected to a phosphopeptide enrichment procedure using titanium dioxide (TiO_2_) beads. The flow-through (nonmodified peptides) and bound (phosphorylated peptides) fractions were desalted on R3 stage tip column and subsequently fractionated by hydrophilic interaction liquid chromatography (HILIC) fractionation. All fractions were analyzed using nLC-MS/MS. Acquired raw MS datasets were processed using Proteome Discoverer three for protein identification and quantification followed by bioinformatics analysis and functional validation. *B*, Venn diagram showing the profile of mouse hippocampal PSD proteome and phosphoproteome identified and quantified from this study. *C*, Venn diagram showing the number of unambiguous phosphosites (75%<phosphosite localization probability), unique phosphopeptide groups, and phosphoPSMs identified from the hippocampal PSD fractions. *D* and *E*, Two-dimensional principal components analysis (PCA) comparing regulated nonmodified (*left*) and phosphorylated proteins (*right*) from IA (*red*), Walk (*green*), Shock (*blue*), and naïve (*brown*) groups based on components 1 and 2, which accounted for 52.1/30.7% for nonmodified proteins and 51.4/31.6% for phosphoproteins of the variability, respectively.

To identify and characterize changes in PSD proteins and their phosphorylation status and potential signaling mechanisms mediated by IA training, we performed quantitative proteomics and phosphoproteomics followed by bioinformatic analysis in mice that underwent IA training (see Experimental Procedures; Fig. 2*A*). Proteins detected in more than two biological replicates were retained for subsequent analysis with various bioinformatic tools. Missing values were evaluated for each PSD iTRAQ channel that showed as low as ≤2.4%/2.8% at the PSM/phosphoPSM levels, respectively (supplemental Fig. S1). From our master dataset, comprising of Naïve, Walk, IA, and Shock groups, we successfully identified a total of 3938 protein groups (at least two peptides including a unique peptide) and 2761 phosphoproteins (at least 75% of phosphosite localization probability) from PSD fractions, resulting in a total of 4968 proteins identified with an overlap of 1731 proteins (Fig. 2*B*). We next analyzed phosphoproteins identified and quantified from the PSD fractions. In the PSD fractions, we identified a total of 8137 unique phosphopeptides carrying 6188 unique phosphosites on 2761 phosphoproteins (Fig. 2*C*), which are listed in supplemental Table S1.

For an overall assessment of proteomic or phosphoproteomic similarities or differences of the four groups (Naïve, Walk, Shock, and IA training), we used principal component analysis (PCA; see Experimental Procedures). In a PCA from the PSD proteome, components 1 and 2, which account for 52.1% and 30.7% of total variability, respectively, clearly segregated each group into four distinct clusters (Fig. 2*D*). The PCA plot derived from the phosphoproteome data showed similar results (Fig. 2*E*). Together with Western blot validation of various synaptic glutamate receptors in the PSD fractions (Fig. 1, *E* and *F*), these results further validate that the proteome and phosphoproteome changes in these groups were clearly segregated, supporting the modulation of proteins and phosphosites upon IA. Based on this finding and the design of experimental groups reflecting exposure to the new environment (*i.e.*, IA chamber), we used the Walk group as a control for comparative analysis between the IA and Shock groups.

### Experience-Dependent Remodeling of the PSD Proteome and Phosphoproteome

We found that PSD levels of AMPA and NMDA receptors increased following IA training, and, albeit to a lesser extent, in the Walk group compared to the Naïve group (Fig. 1, *E* and *F*). We hypothesized that the experience of exploring the inhibitory avoidance chamber, even in the absence of a shock (and associative emotional-learning), was enough to change neuronal activity similar to environmental enrichment (36, 37, 49) and likely induce some changes in synaptic plasticity. Therefore, to better isolate learning-induced changes, we used the Walk group (with experience of the IA testing chamber) as an internal control in subsequent analyses, thereby comparing the abundance of proteins and phosphoproteins from IA and Shock groups to those from the Walk group.

**Fig. 3:**
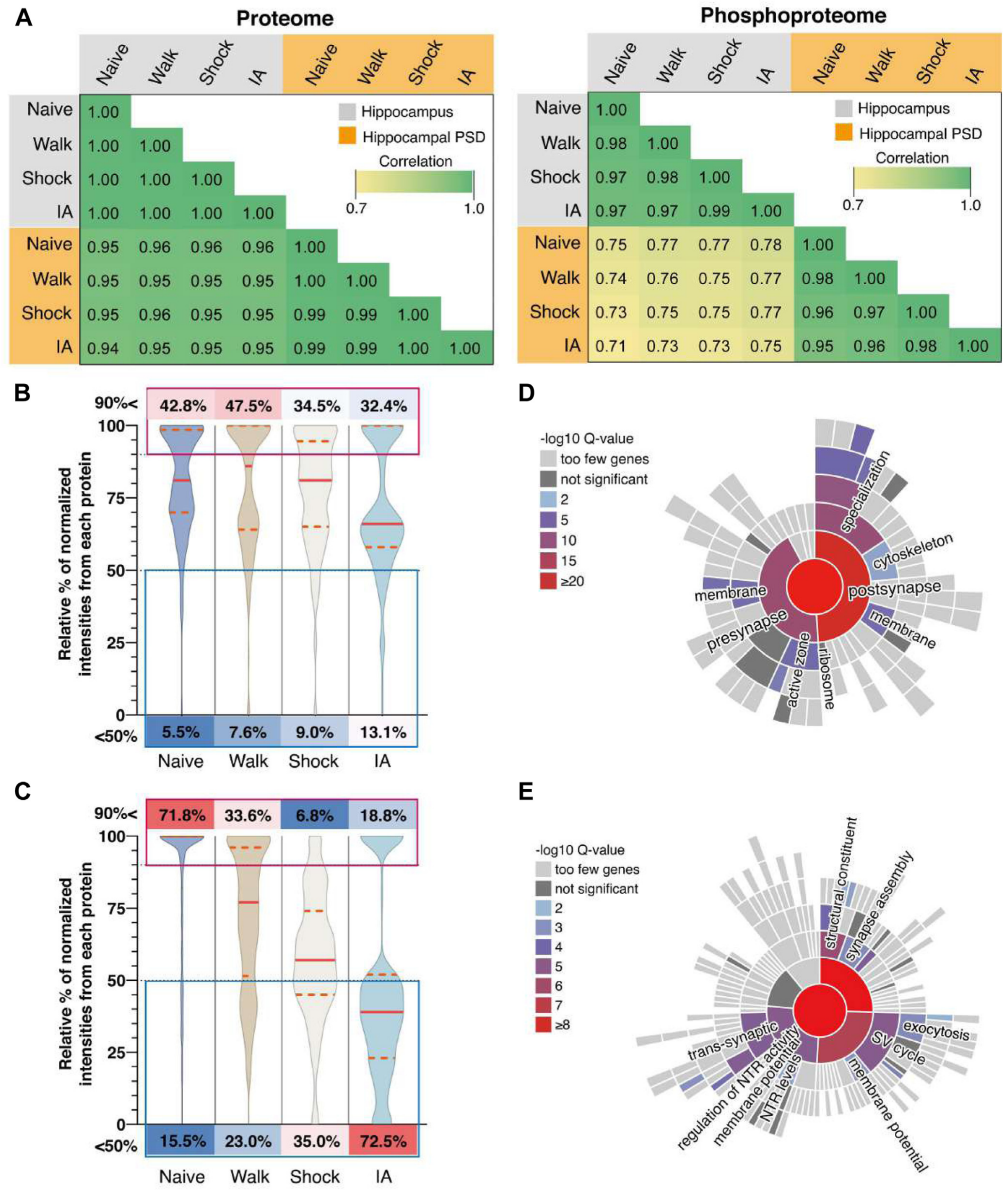
**Dynamic changes of PSD proteins following IA-training and immediate shock**. *A*, correlation matrix of normalized intensities (Log_2_) showing degree of proteome (*left*) or phosphoproteome (*right*) identifications between each sample group and each sample type (*gray*: hippocampus, orange: hippocampal PSD). Pearson correlation coefficient (*r*^2^ range: 0.7–1.0) was calculated among individual proteins or phosphopeptides. Color is scaled by r-squared factor in individual squares. *B*, Violin plot showing the proportion of the percentage of the significantly regulated protein based on normalized quantities in four groups. Y-axis represents the proportions of intensities of the regulated proteins intensities per group. *Red lines* inside the violin plots represent the median of overall percentages per group. The width of the plot represents the density of proteins. Interquartile ranges are marked with orange dashed lines (Q3: *upper quartile*, Q1: *lower quartile*). *C*, violin plot showing the proportion of the significantly altered phosphorylation percentage based on normalized quantities in four groups. Y-axis represents the proportions of intensities of the altered site-specific phosphorylation intensities per group. Red lines inside of the violin plots represent the median of overall percentages per group. The width of the plot represents the density of site-specific phosphorylation. Interquartile ranges are marked with *orange dashed lines* (Q3: *upper quartile*, Q1: *lower quartile*). *D*, interactive heat map of the experience-dependent regulatory proteins and phosphosites showing the categories of synaptic subcellular locations obtained by Synaptic Gene Ontologies (SynGO) annotations. The probability score based on −log10 Q-value of synaptic location annotation are represented. *E*, interactive heat map of the experience-dependent regulatory proteins and phosphosites showing the categories of synaptic biological functions obtained by Synaptic Gene Ontologies (SynGO) annotations. The probability score based on −log10 Q-value of synaptic function annotation is represented. NTR, neurotransmitter receptor; SV, synaptic vesicle.

To comprehensively evaluate all the proteins and phosphopeptides for differences in hippocampus and subcellular PSD fraction and between each group and all other groups, correlation analysis was conducted (Fig. 3*A*). In the proteome data, Pearson correlation coefficients of >0.99 were observed between all the experiment groups, and all PSD fractions were equivalent to each hippocampus (.94 < *R*^2^). Conversely, the phosphopeptides for the PSD groups were relatively less correlated with the hippocampus groups (*r*^2^ range: .71−.78), implying a larger variation at the phosphorylation level in their enrichment for PSD compared to the total hippocampus.

Next, we hypothesized that IA training or immediate shock would comprise changes, especially in the phosphorylation level of synaptic proteins that perform critical synaptic functions. To assess this, we analyzed the overall changes of the proteins and site-specific phosphorylation degrees in the PSD (phospho)proteome in IA and Shock groups compared to the Walk group. We observed that PSD proteins (n = 145) and site-specific phosphorylation (n = 309) were regulated following IA training (adjusted *p*-value < 0.05); these are included in supplemental Table S1. We next analyzed the trend of changes in expression and phosphorylation levels in the hippocampal PSD fractions following IA training or immediate shock. The degree of alterations of all regulated proteins showed insignificant changes in all four groups (Fig. 3*B*). The decrease in protein levels is more obvious in the IA group, indicating that this decrease is somewhat task-specific. Interestingly, we observed that overall phosphorylation levels showed a decreasing trend in Shock and IA groups compared to the Walk group, with the IA group showing the greatest decrease in phosphorylation levels (Fig. 3*C*). The overall phosphorylation decreases of approximately 45% were observed in the median of Shock group compared to the median of Naïve group. In comparison, in the median of the IA group phosphorylation decreases of approximately 70% were observed 1-h post-training, while the phosphorylation decrease was only around 25% in the Walk group. Our finding suggests that decreased phosphorylation may play roles in experience-dependent synaptic plasticity and learning and memory formation at early time points (∼1 h). However, it remains to be established whether the decrease in phosphorylation level in the PSD is reflecting decreased protein levels, or if other molecular mechanisms (*e.g.*, inactivation of kinases or activation of phosphatases) are involved.

Proteins and phosphoproteins regulated by IA training were defined by synaptic Gene Ontology annotation (synGO) analysis to reveal enriched subcellular locations (Fig. 3*D*) and biological functions (Fig. 3*E*). As shown in Fig. 3, *B* and *C*, we observed an overall reduction of protein and phosphorylation levels in the IA and Shock group. However, it should be noted that the function of individual phosphosites often is not known and therefore, the functional validation of selected phosphosites will be required to uncover the potential roles in synaptic plasticity or learning and memory. Analysis of synaptic subcellular locations belonging to IA-regulated proteins and phosphoproteins revealed significant enrichment of protein localization mostly involved in postsynaptic specialization, cytoskeleton, membrane, and ribosome (Fig. 3*D*). Analysis of synaptic biological functions showing belonging to IA-regulated proteins and phosphoproteins revealed significant enrichment of biological process terms, such as structural constituent, synaptic assembly, exocytosis, synaptic vesicle cycle, membrane potential, neurotransmitter receptor levels, regulation of neurotransmitter receptor activity, and trans-synaptic process (Fig. 3*E*). Taken together, levels of proteins and their phosphosites are dynamically regulated by the different types of experience.

### Bioinformatic Analysis of Experience-Dependent Proteome and Phosphoproteome Dynamics

**Fig. 4:**
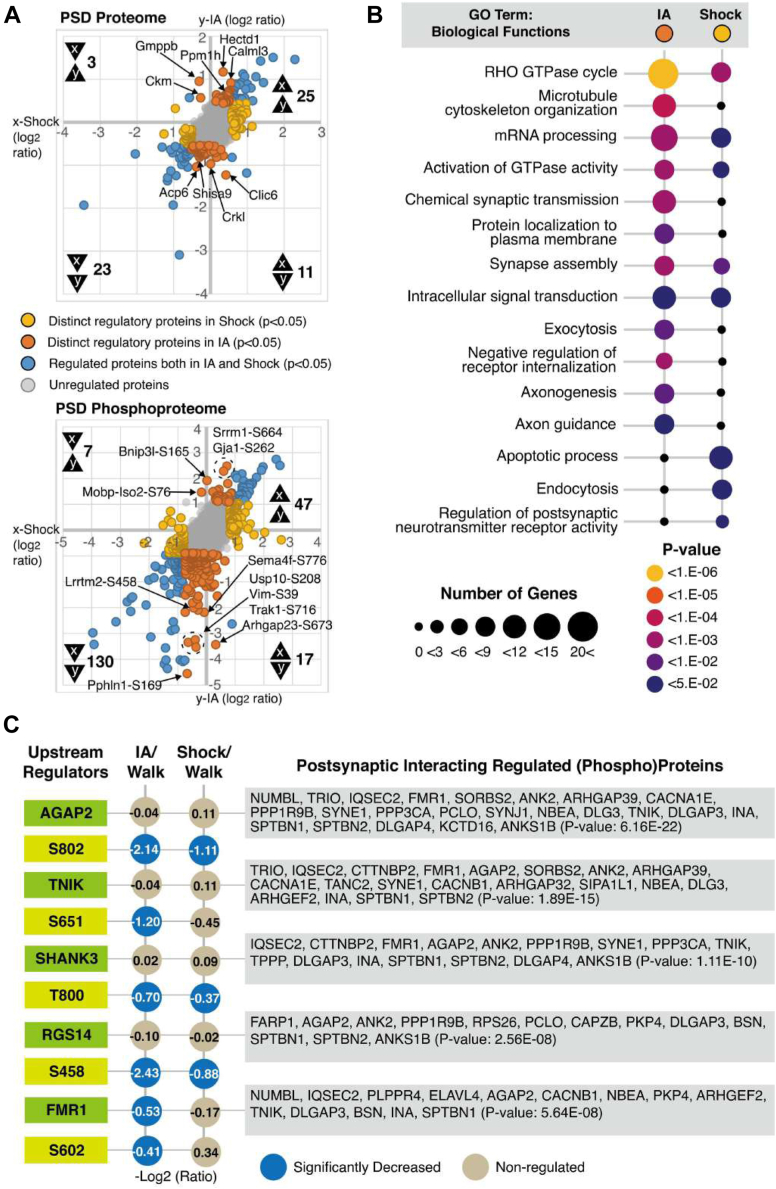
**Bioinformatics analysis of PSD proteins and phosphoproteins regulated by IA-training and immediate shock.***A*, scatter plot showing all proteins (*left panel*) and phosphopeptides carrying relevant phosphosites in IA (x-axis) or Shock (y-axis) group compared to Walk group. Proteins and phosphosites that were significantly regulated by IA training or immediate shock are indicated in blue. Proteins and phosphosites that were significantly regulated by IA are indicated in orange. Proteins and phosphosites that were significantly regulated by immediate shock are indicated in *yellow*. Other proteins and phosphosites that were not regulated are displayed in *gray*. Proteins and phosphopeptides showing same- (IA↑/Shock↑ or IA↓/Shock↓) or bi-directional (IA↑/Shock↓ or IA↓/Shock↑) regulations are indicated in each quadrant. *B*, gene ontology (GO) enrichment analysis of regulated proteins and phosphoproteins in IA or Shock groups showing the number of genes and the *p*-value for the indicated groups. DAVID Gene Ontology analysis including Reactome pathway database was performed to show enriched biological functions (*p*-value < 0.05). *C*, predicted upstream regulator interacting with postsynaptic significantly regulated (phospho)proteins using BioGRID database in DAVID search.

To analyze individual unique proteins and their phosphosites regulated by IA training or immediate shock, we grouped proteins and phosphoproteins based on their direction of change compared to the Walk group. We discovered 62 unique protein groups that were significantly regulated following IA training or immediate shock (Fig. 4*A* upper panel, IA↑/Shock↑: 25, IA↓/Shock↓: 23, IA↑/Shock↓: 3, IA↓/Shock↑: 11) and 201 unambitious phosphosites (Fig. 4*A* lower panel, IA↑/Shock↑: 47, IA↓/Shock↓: 130, IA↑/Shock↓: 7, IA↓/Shock↑: 17). Among these regulated proteins, we identified 14 proteins and 24 phosphosites distinctively regulated by IA training. Figure 4*A* depicts all proteins and phosphosites analyzed, and each quadrant in the graphs shows four different categories of changes in the expression or phosphorylation levels of individual proteins. The upper right and lower left quadrants depict proteins and phosphoproteins that exhibit the same directional regulation: both IA training and immediate shock result in either an increase (upper right quadrant, 25 proteins and 47 phosphosites) or a reduction (lower left quadrant, 23 proteins and 130 phosphosites) in the expression or phosphorylation levels (Fig. 4*A* and supplemental Table S2) when compared to the Walk group. The remaining categories are represented by proteins and phosphoproteins regulated in a bi-directional manner. Enhanced protein or phosphorylation levels for these proteins are associated with one form of experience (either IA training or immediate shock) while reduced protein or phosphorylation levels are associated with the other form of experience. Proteins and phosphoproteins categorized in upper left quadrant (3 proteins and seven phosphosites) were enhanced by IA training but reduced by immediate shock while those in the lower right quadrant (11 proteins and 17 phosphosites) were reduced by IA training but enhanced by immediate shock (Fig. 4*A* and supplemental Table S3). Taken together, we found dynamic remodeling of the PSD proteome and phosphoproteome following IA training or immediate shock compared to the walk-through control.

To isolate changes unique to IA training or immediate shock, we looked for proteins and phosphoproteins that were regulated distinctively following IA training but not immediate shock or vice versa. Proteins and phosphosites regulated by a single factor exclusively (IA training or immediate shock) are represented as spots close to x- (shock-unique) or y-axis (IA-unique) on the scatter plot shown in Figure 4*A*. DAVID GO analysis (incl. Reactome pathway database) of proteins and phosphoproteins regulated distinctively by IA training indicates a significant enrichment for proteins involved largely in the regulation of synaptic functions including RHO GTPase cycle, microtubule cytoskeleton organization, mRNA processing, activation of GTPase activity, chemical synaptic transmission, protein localization to plasma membrane, synapse assembly, intracellular signal transduction, exocytosis, negative regulation of receptor internalization, axonogenesis, and axon guidance (Fig. 4*B* and supplemental Table S4). In the case of proteins and phosphoproteins uniquely regulated by immediate shock, GO analysis of this group indicates significant enrichment of cellular functions involved in the apoptotic process, endocytosis, and regulation of postsynaptic neurotransmitter receptor activity (Fig. 4*B* and supplemental Table S4). Taken together, both IA training and immediate shock seem to engage some overlapping cellular functions, but there are also proteins and phosphoproteins that are regulated uniquely by IA training or immediate shock, which show distinct biological functions in the GO analysis.

Next, we conducted protein-protein interaction analysis using BioGRID database in DAVID search to unveil upstream modulators shared by the list of regulated proteins and phosphoproteins following IA training in the PSD (Fig. 4*C*). Five predicted upstream regulators (AGAP2-S802, TNIK-S651, SHANK3-T800, RGS14-S458, and FMR1-S602) showed high confidence (FDR ≤ 5.64E-08), indicating that were significantly dephosphorylated in IA training mediated by postsynaptic interacting regulated (phospho)proteins (Fig. 4*C*).

### Clustering and Mapping of Protein Interaction Network Related to IA-Learning

**Fig. 5:**
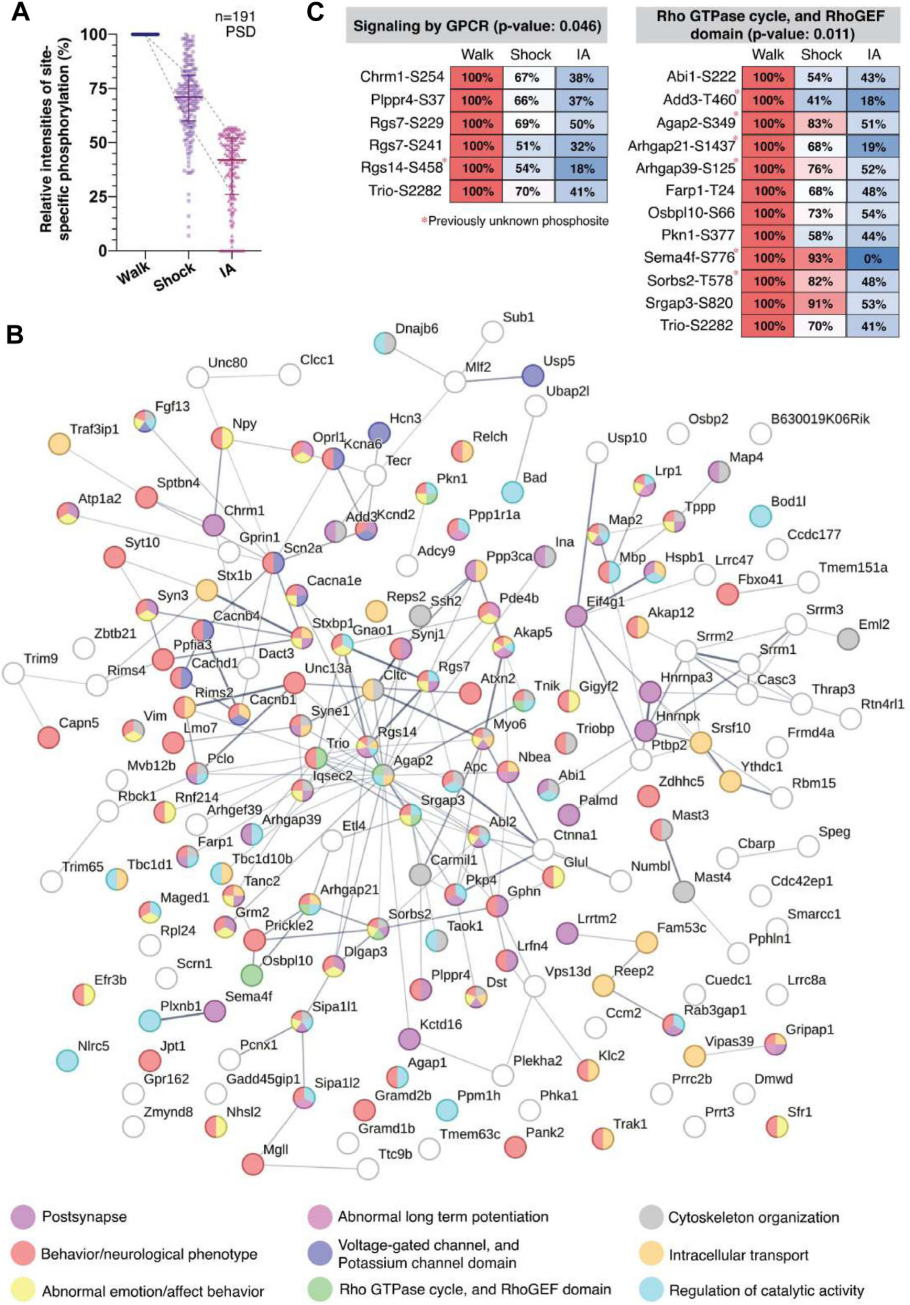
**Protein–protein interaction network of regulated PSD phosphopeptides carrying relevant phosphosites following IA-training and immediate shock.***A*, most enabled clustering map of regulated phosphoproteins carrying relevant phosphosites that significantly decreased following IA-training (n = 191, adjusted *p*-value < 0.05). *B*, Protein–protein interaction networks of the hypophosphorylated proteins carrying relevant phosphosites show a decreasing pattern following IA training. The phosphoproteins from the most enabled cluster (significantly decreased in IA) were analyzed against STRING database to generate an interaction network map (confidence score >0.4; medium confidence). The biological function of each gene is defined by DAVID GO analysis and colored by the association with each GO term. *C*, Overrepresented two biological contexts (*p*-value < 0.05) of the interconnected PSD phosphoproteins with their relevant phosphorylation sites significantly decreased in IA. The quantitative information for each phosphoprotein is shown as relative concentration (0–100%) in a box format together with the information on designated phosphorylation sites. *Asterisks* indicate previously unreported phosphosite in UniProt database.

Next, we performed clustering analysis to characterize the most enriched clustering patterns among differentially modulated site-specific phosphorylation following IA training. We found that 191 regulated phosphoproteins were clustered in the group which decreased in the IA group compared to the Walk group (Fig. 5*A*).

We next used a combined approach of STRING (https://string-db.org/), DAVID (https://david.ncifcrf.gov/), and Reactome (https://reactome.org/) pathway analyses on the large-scale dataset to systematically reveal protein-protein interaction networks among significantly regulated phosphoproteins (35). We found that significantly regulated phosphoproteins from PSD fractions in this cluster were closely connected (Fig. 5*B*). We also found that relationship and individual molecular interactions of altered phosphoproteins enriched nine biological processes, including postsynapse, behavior/neurological phenotype, abnormal emotion/affect behavior, abnormal LTP, voltage-gated channel, and potassium channel domain, RHO GTPase cycle, and RhoGEF domain, cytoskeleton organization, intracellular transport, and regulation of catalytic activity, which are all associated with synaptic functions (Fig. 5*B*).

Within the postsynaptic compartment, we found overrepresented two biological functions (signaling by GPCR and RHO GTPase cycle, and RhoGEF domain where IA training is associated with the reduction of phosphorylation (Fig. 5*C*). This result suggests that roles of these phosphoproteins in signaling pathways are associated with experience-dependent remodeling of the synaptic proteome in the hippocampus after IA training.

### Identification of Regulated Kinases and Phosphatases

Protein phosphorylation is one of the most common PTMs, controlling important cellular processes through the action of kinases and phosphatases. Neuronal plasticity which mediates learning and memory also requires different kinases and phosphatases that can reversibly phosphorylate and dephosphorylate specific sites on target proteins (14, 16, 50). As shown in Figures 3 and 4, we observed dynamic changes in phosphorylation on several proteins, including various kinases and phosphatases, from the hippocampal PSD fraction. We performed sequence homology analysis (see Experimental Procedures) of the significantly regulated kinases and phosphatases to characterize the regulation pattern of individual enzymes and to identify novel kinases and phosphatases and their phosphosites associated with IA training.

**Fig. 6:**
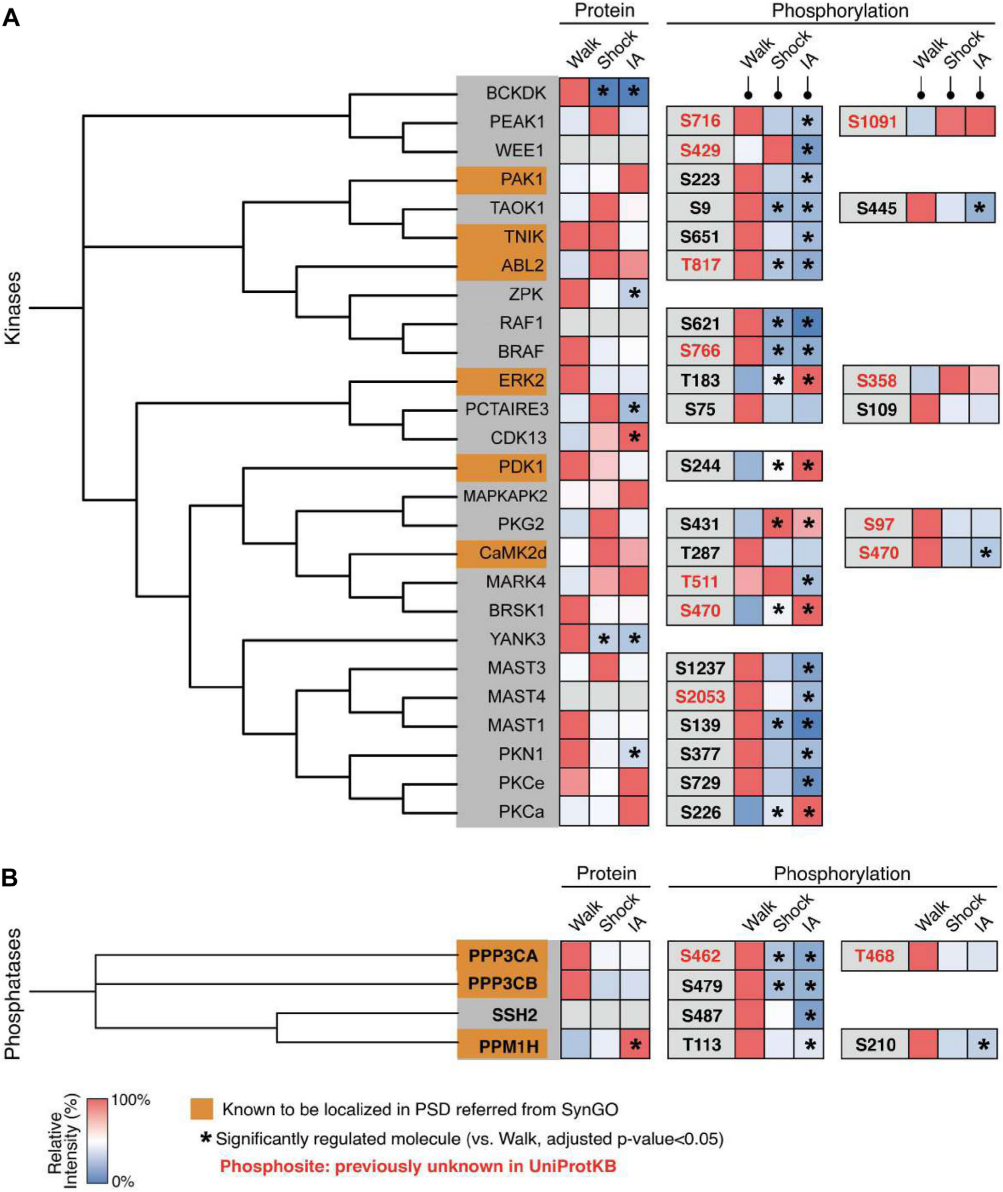
**Profiling of regulated protein kinases and phosphatases.***A*, Kinome analysis of the significantly regulated kinases (*A*) and phosphatases (*B*) at the protein and phosphorylation levels in the PSD following IA training or immediate shock with their homology of the kinase domains. Kinases that are known to exist in the PSD are labeled in *orange*. Relative intensities (%) of significantly regulated kinases following IA training or immediate shock are shown in color-coded boxes (inside of the *gray rim*). The levels (log_2_ ratio) of phosphorylation of specific residues on individual kinases are shown as a circular index with different colors (*green*: up-regulated, *blue*: down-regulated) and size (outside of the *gray rim*). Outer and inner circles indicate Log_2_ ratio of given phosphosites in IA and Shock groups compared to Walk group, respectively.

We found that 26 kinases were significantly regulated in the IA training group (Fig. 6*A*). These include six kinases, such as Pak1, Tnik, Abl2, Erk2, Pdk1, and CaMK2d, which are already known to be localized in the PSD and play crucial roles for synaptic plasticity (51, 52, 53, 54, 55, 56, 57). CaMK2 functions as homomeric or heteromeric holoenzyme complexes, and each 12 subunits have different roles in synaptic plasticity (58). The expression of PSD CaMK2d protein was not obviously changed in both the IA and Shock groups with the most relative increase in the Shock group. The kinase phosphorylation pattern was differentially regulated compared to the total protein levels by different experiences. For example, CaMK2d phosphorylation on S470 (previously unreported phosphosite) was significantly decreased, while the total CaMK2d protein level in the hippocampus was significantly increased in the IA group (adjusted *p*-value: 0.0384). Similarly, the expression of Pak1, Abl2, Mark4, and Prkce (also known as PKCe) proteins was increased, whereas their phosphorylation levels at S223, T817 (new phosphosite), T511 (new phosphosite), and S729 were significantly decreased in IA group, respectively. Besides, the total Erk2 and Pdk1 protein levels in PSD were decreased, while phosphorylation on Erk2-T183, Erk2-S358 (previously unreported phosphosite), and Pdk1-S244 were highly increased in both IA and Shock groups. A series of kinases including Bckdk, Zpk, Pctaire3, Yank3, and Pkn1 was significantly reduced at protein level in the IA group compared to the Walk group. Prkca (also known as PKCa) showed increasing expression and phosphorylation (S226) levels in both the IA group (Fig. 6*A*). Our results show that the expression patterns of different kinases are divergent depending on the experience.

The levels of regulated phosphatases also showed varied patterns. We identified four phosphatases (Ppp3ca, Ppp3cb, Ssh2, and Ppm1h), which were significantly regulated in the IA group (Fig. 6*B*). Three phosphatases, Ppp3ca (also known as PP2BA), Ppp3cb (also known as PP2BB), and Ppm1h, are known to be localized in the PSD and act important roles for synaptic plasticity (59, 60, 61). We also identified phosphorylations of these phosphatases which decreased following IA group (Ppp3ca (S462 and T468), Ppp3cb (S479), Ssh2 (S487), and Ppm1h (T113 and S210)) (Fig. 6*B*). Taken together, these results show that the expression levels of kinases and phosphatases and their phosphorylation levels are regulated differentially by experience.

### Dephosphorylation of Ppm1h Protein Induced by IA Training

**Fig. 7:**
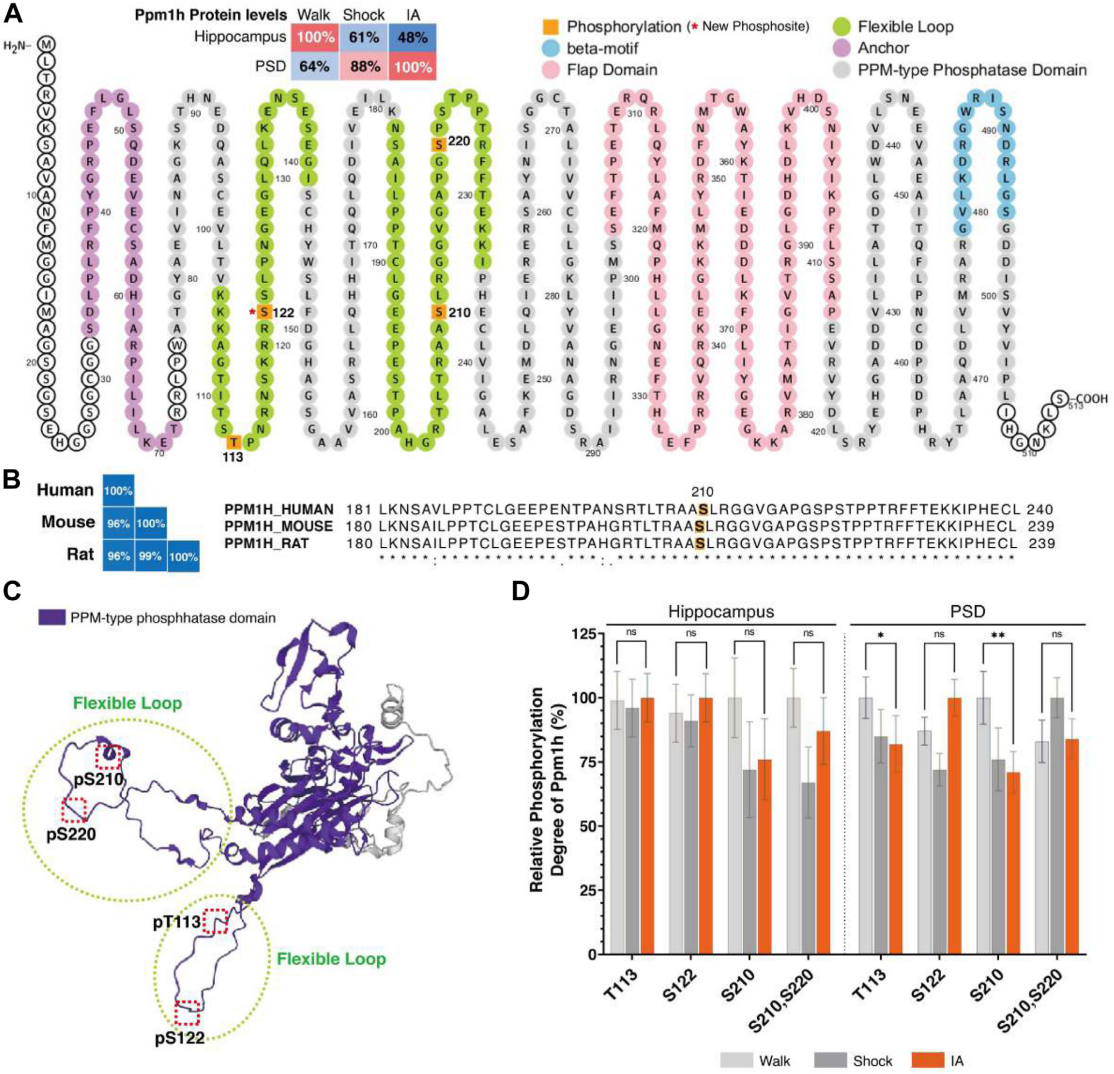
**Mapping of Ppm1h protein, phosphorylation, and structure.***A*, experience-dependent regulation of Mg^2+^/Mn^2+^-dependent protein phosphatases (Ppm1h). Clustered isoforms of Ppm family showed unique regulations. After IA training, the expression of protein Ppm1h was the highest in PSD, but it was the lowest expression in hippocampi. *B*, taxonomy and protein sequence homology of mouse Ppm1h identity (%) esteemed by sequence alignment evaluation using Clustal Omega (*left*). Sequence alignment of the flexible loop of PPM1H (*right*) that contains the phosphorylation at S210 (*orange squares*). *C*, 3D structure of Ppm1h protein visualized by AlphaFold structure database. *D*, the phosphorylation levels on Ppm1h (T113 and S210) showed significant decrease following IA-training (n = 3/group; adjusted *p*-value for Walk vs. IA, ∗*p* ≤ 0.05 and ∗∗*p* ≤ 0.01 by Benjamini multiple *t* test).

We observed a noticeable pattern of phosphorylation decrease in both the IA and Shock groups compared to Naïve or Walk groups (Fig. 3*C*). This finding led us to speculate about the roles of protein phosphatases during IA training-mediated learning or in response to immediate shock. From our dataset, levels of protein phosphatases and their phosphorylation were found to be differentially regulated following IA training. Within the set of protein phosphatases regulated by IA training or immediate shock, Ppm1h protein expression, previously reported to be linked to synaptic plasticity (60, 61), was decreased in the total hippocampal lysate following IA training, but it was increased in PSD fractions (Fig. 7*A*), suggesting that could potentially implicate translocation to PSD mediated by IA training. Within Ppm1h protein phosphatase regulated by IA training, we identified that phosphorylation of Ppm1h at T113, S122, S210, and S220 in flexible loops (Fig. 7*A*). Multiple protein sequence alignments of Ppm1h were achieved by Clustal Omega, showing homologous species groups that shared 96% or higher sequence similarity across human, mouse, and rat species (Fig. 7*B*). Interestingly, all the phosphosites were detected within flexible loops, which were visualized by AlphaFold structure database (Fig. 7*C*). Notably, the phosphorylation at T113 and S210 were significantly down-regulated (*p*-value < 0.05) in the IA training group when compared to Walk group in PSD fractions (Fig. 7*D*).

### Functional Validation of Ppm1h Regulated by IA Training or Immediate Shock

**Fig. 8:**
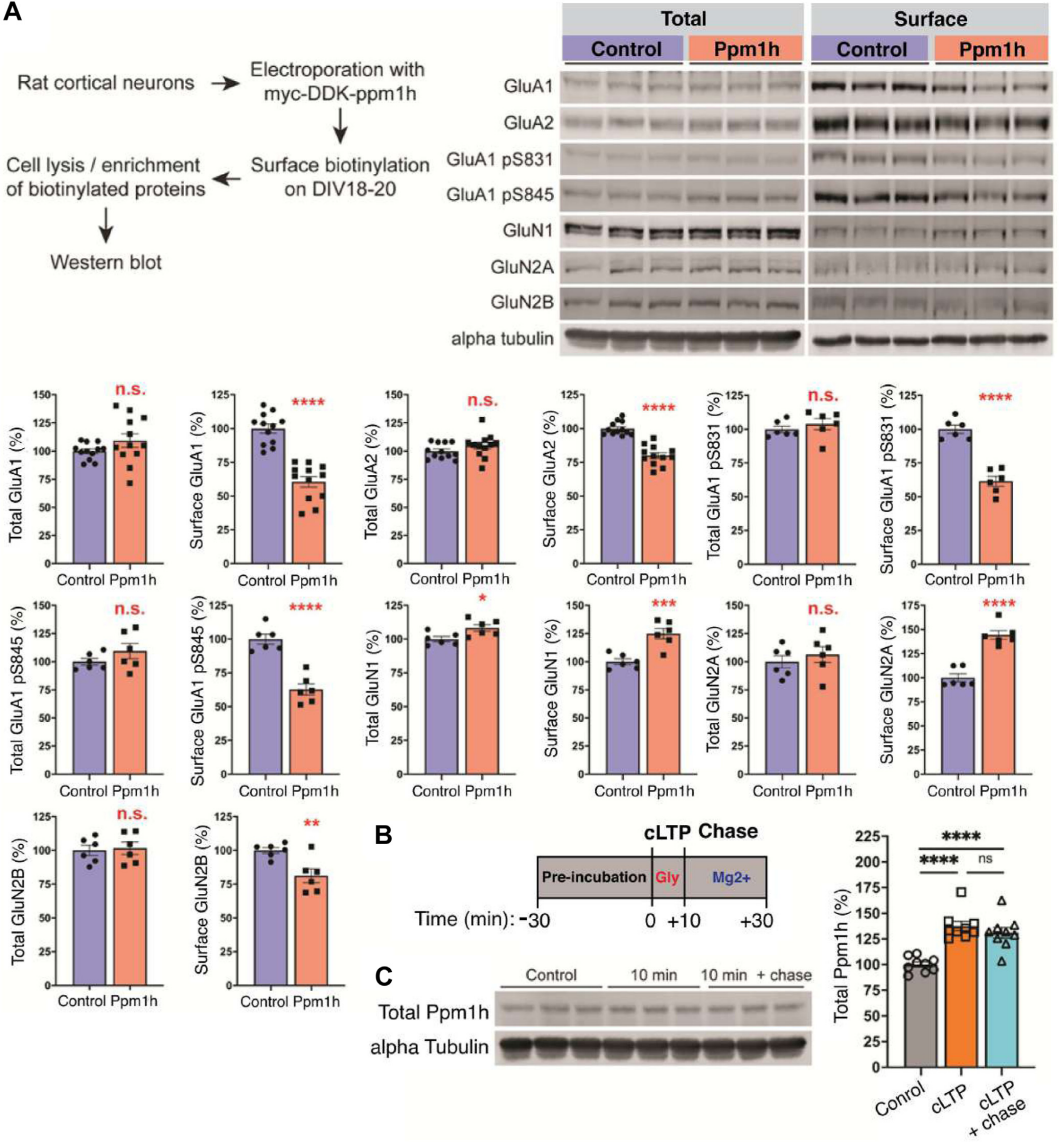

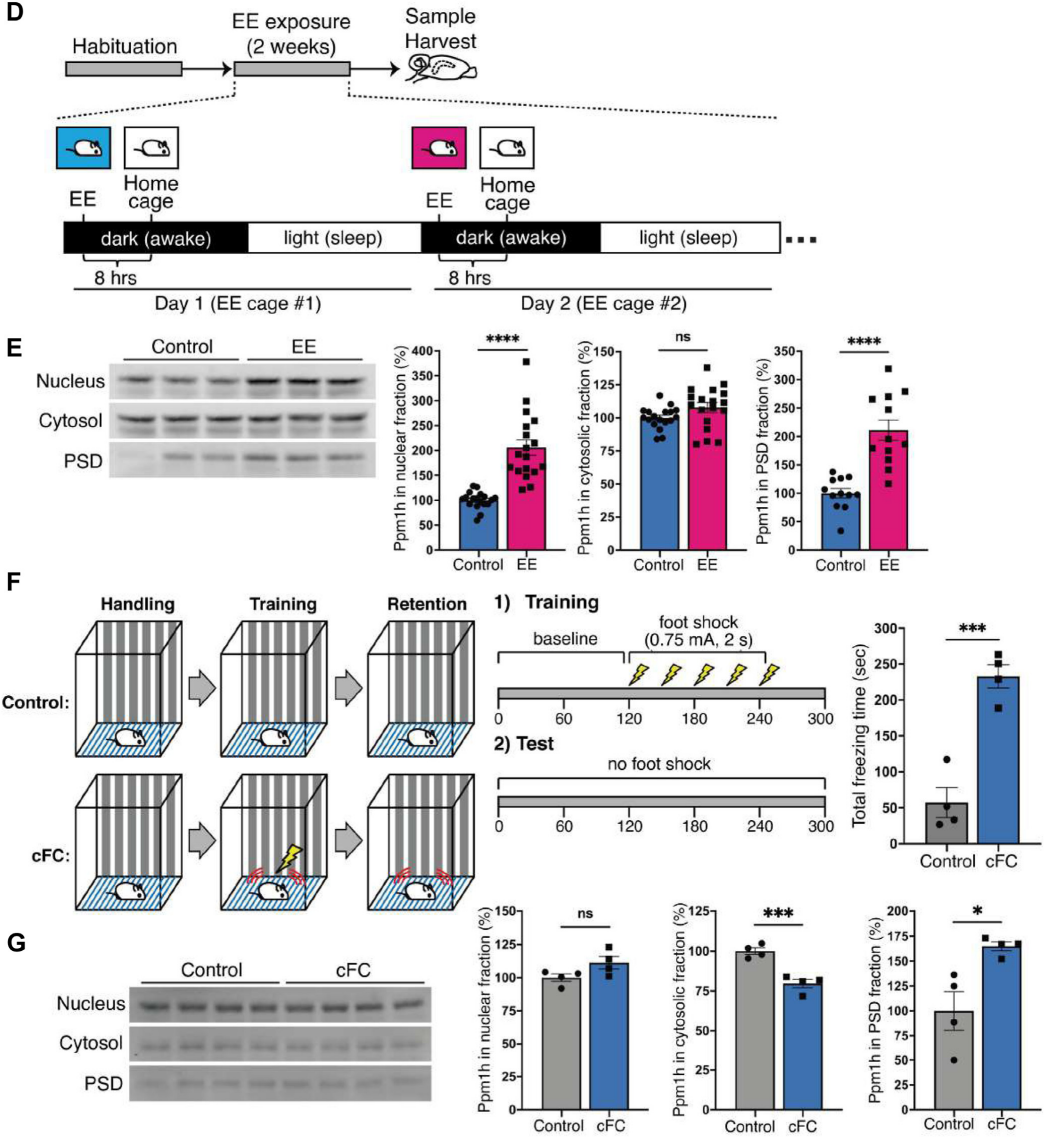
**Functional validation of Ppm1h in synaptic plasticity**. *A*, Differential regulation of surface expression of AMPAR and NMDAR subunits by Ppm1h overexpression. When Ppm1h was overexpressed, cortical neurons exhibited increased surface expression of GluN1 and GluN2A. In contrast, surface expression of GluA1, A2, GluA1 pS831, pS845 and GluN2B showed a significant decreased following Ppm1h overexpression (n = 12 for GluA1 and GluA2, and six for other glutamate receptors; ∗*p* ≤ 0.05, ∗∗*p* ≤ 0.01, ∗∗∗*p* ≤ 0.005, and ∗∗∗∗*p* ≤ 0.001 by Student’s *t* test). *B* and *C*, regulation of Ppm1h during glycine-induced chemical LTP (GI-cLTP). *B*, scheme indicating experimental workflow for GI-cLTP. Cultured cortical neurons (DIV18 or older) were treated with 200 μM glycine for 10 min followed by chase with Mg^2+^-containing ACSF for 30 min. *C*, GI-cLTP increased the expression level of Ppm1h levels after both 10 min stimulation and 30 min chase (n = 9/group; adjusted *p*-value for Control vs. 10 min and Control vs. 30 min chase, ∗*p* ≤ 0.05, ∗∗*p* ≤ 0.01, and ∗∗∗*p* ≤ 0.005 by 1-way ANOVA). *D* and *E*, effect of enriched environment (EE) exposure to the level of Ppm1h in the mouse hippocampus. *D*, scheme indicating experimental workflow for exposure to EE. Mice were tested for 2 weeks using a 2-day EE exposure paradigm (see Experimental Procedures), followed by isolation of subcellular fractions (nuclear, cytosolic, and PSD fractions) from the hippocampus. *E*, characterization of Ppm1h expression in different subcellular fractions following EE exposure. EE resulted in a significant increase of Ppm1h in the nucleus and PSD while cytosolic Ppm1h levels did not change (n = 18/group for P1 and PS2 fractions, n = 12 for PSD fractions; ∗∗∗∗*p* ≤ 0.001 by Student’s *t* test). *F* and *G*, effect of contextual fear conditioning (cFC) to the level of Ppm1h in the mouse hippocampus. *F*, scheme indicating experimental workflow for cFC. Mice were trained *via* cFC task (see Experimental Procedures), followed by isolation of subcellular fractions (nuclear, cytosolic, and PSD fractions) from the hippocampus. Mice showed a significant increase in time spent freezing after cFC (n = 4/group; adjusted *p*-value, ∗∗∗*p* < 0.005 by 2-way ANOVA). *G*, characterization of Ppm1h expression in different subcellular fractions following cFC. Ppm1h significantly increased in PSD fractions while cytosolic or nuclear Ppm1h levels showed a significant decrease or no change after cFC training, respectively (n = 4; ∗*p* ≤ 0.05, ∗∗*p* ≤ 0.01, and ∗∗∗*p* ≤ 0.005 by Student’s *t* test).

We examined whether Ppm1h can be affected by different types of neuronal activity or regulate synaptic plasticity *in vitro* and *in vivo*. First, we electroporated Ppm1h into cultured neurons and monitored the effect of Ppm1h overexpression on the levels of surface AMPA and NMDA receptors. Following Ppm1h overexpression, we observed a decrease of surface GluA1, GluA2, and phospho-GluA1 at S831 and S845, but no obvious changes in total expression level (Fig. 8*A*). Interestingly, Ppm1h overexpression resulted in an increase of surface NMDAR subunits, GluN1 and GluN2A, but a decrease of surface GluN2B, indicating that Ppm1h likely regulates AMPARs and NMDARs differentially (Fig. 8*A*). This result suggests that Ppm1h may be involved in regulating surface expression of AMPA and NMDA receptors however, detailed molecular mechanisms remains unclear. Next, we performed glycine-induced chemical LTP in cultured neurons and examined the abundance of Ppm1h (Fig. 8*B*). The total Ppm1h level increased after 10 min glycine stimulation and was maintained during the 30 min chase period in the presence of Mg^2+^ (Fig. 8*C*), suggesting that Ppm1h levels can also be regulated by chemical LTP.

Next, we investigated the regulation of Ppm1h following neuronal activity changes *in vivo*. First, we exposed mice to an EE to induce brain-wide changes in neuronal activity and examined the levels of hippocampal Ppm1h from different subcellular fractions (Fig. 8*D*; see Experimental Procedures). We observed increased levels of Ppm1h from nuclear and PSD fractions while cytosolic fraction did not show any significant changes in the hippocampus after EE exposure (Fig. 8*E*). Second, we similarly examined the levels of hippocampal Ppm1h from different subcellular fractions after cFC to examine the effect of learning-specific neuronal activity changes (Fig. 8*F*; see Experimental Procedures). Interestingly, we observed a significant decrease of Ppm1h in the cytosol and an increase in PSD fractions while nuclear Ppm1h levels did not change (Fig. 8*G*). Taken together with our quantitative proteomic results, these data indicate that levels of Ppm1h are differentially regulated by different types of neuronal activity and that manipulation of Ppm1h level can affect the trafficking of glutamate receptors to the surface of synapses, which may subsequently affect the regulation of synaptic plasticity *in vitro* and *in vivo*. This places de-phosphorylation as another important regulator of synaptic plasticity.

## Discussion

In this study, we developed a new strategy to identify and quantify the relatively low signals of site-specific phosphorylation of postsynaptic density (PSD) phosphoproteins from the hippocampus, located in the medial temporal lobe of the mouse brain, responsible for managing cognition and memory (1, 2). By using a modified iTRAQ-based TiSH protocol (33, 34, 35), phosphopeptides were detected *via* MS/MS scans. Accordingly, we identified and quantified 3938 hippocampal PSD protein groups and 2761 phosphoprotein groups (including 6188 unique phosphosites). Considerable modulations in the expression levels of PSD proteins and their site-specific phosphorylation were observed after inhibitory avoidance (IA) training or immediate shock. These alterations were largely involved in neuronal functions, such as synaptic plasticity, regulation of neurotransmitter receptors, ion channels, and structural organization of synapses. We provide comprehensive datasets highlighting experience-dependent remodeling of the hippocampal PSD-specific proteome and phosphoproteome *in vivo*. The derived lists of hippocampal PSD proteins and their site-specific phosphorylation from our quantitative proteome and phosphoproteomics analyses represent the dynamic patterns of hippocampal postsynaptic signaling that may shed new light on the mechanisms underlying synaptic plasticity and learning and memory.

We report dynamic changes of the PSD-specific proteome and phosphoproteome in the mouse hippocampus dissected from individual mice following four different types of experience to investigate molecular mechanisms underlying experience-dependent remodeling of synapses. We applied multiple control groups, including the Naïve group, to distinguish the effect of learning on proteome remodeling by excluding the effect of other external stimuli, such as exposure to the new environment (Walk) or immediate aversive stimulation (Shock). Correspondingly, we found that hippocampal PSD proteins and their phosphorylation levels are dynamically modulated following the robust learning mediated by IA training compared to Shock or Walk. Here, we employed the IA task which is a commonly used behavioral task to investigate learning and memory processes (5, 7, 8, 62). This task consists of a single training session and a subsequent recall test to assess memory formation. While the task is simple, the underlying mechanisms for memory acquisition, consolidation, storage, and retrieval are complex. Here, we set up a group of mice in which foot shock was delivered immediately after exposure to the IA training chamber to distinguish IA training-induced proteome and phosphoproteome dynamics from a shock-only stress response. A general question is whether there are proteins that play overlapping or distinctive following IA training and/or immediate shock. GO analysis of proteins regulated by IA training revealed a series of cellular functions that were significantly enriched both in IA and Shock group, or enriched uniquely in either IA or Shock group. We also identified a series of cellular functions that were uniquely enriched in either the IA or Shock group. For example, cellular functions, such as RHO GTPase cycle, microtubule cytoskeleton organization, mRNA processing, activation of GTPase activity, chemical synaptic transmission, protein localization to plasma membrane, synapse assembly, intracellular signal transduction, exocytosis, negative regulation of receptor internalization, axonogenesis, and axon guidance, were distinctively enriched in IA group (Fig. 4*B*). In the same way, we found that cellular functions including apoptotic process, endocytosis, and regulation of postsynaptic neurotransmitter receptor activity were uniquely enriched in Shock group (Fig. 4*B*). Proteins that exhibit same cellular functions in both IA and Shock groups may be categorized to common proteins that respond to various experiences. On the other hand, proteins linked to cellular functions distinctively enriched in either the IA or Shock group may be categorized by unique proteins that respond differentially to either IA training or immediate shock. These findings support the idea that those behavioral phenotypes elicited by different forms of experience (here IA training and immediate shock) are mediated by proteins or PTMs involved in (1) shared cellular functions that can be regulated in either the same or different directions and (2) unique cellular functions that are differentially enriched following specific experiences. Functional validation of PSD proteins or phosphosites that are uniquely regulated by IA training or immediate shock will be required to better understand molecular mechanisms for learning and memory formation.

Synaptic plasticity is associated with the delivery of different types of glutamate receptors to the synapses (1). In a previous study, phosphorylation of GluA1 at Ser831 increased, whereas phosphorylated GluA1 at Ser845 was not affected by IA training. Synaptic targeting of total GluA1 and GluA2 AMPA receptor subunits, but not NR1 NMDA receptor subunits, was enhanced after IA training (5). Our validation experiment showed a net increase of GluA1, GluA2, two well-characterized phosphosites of GluA1 (Ser831 and Ser845), and NMDAR subunits (GluN1, 2A, 2B) in PSD fraction after IA training (Fig. 1, *E* and *F*). Interestingly, we observed a significant increase of AMPAR, NMDAR subunits, and two well-characterized phosphosites of GluA1 in the mouse hippocampal PSD fractions from the Walk group compared to the Naïve group. Our approach is a short-term exposure to the training chamber (duration: maximum 5 min, number of exposures: maximum two times) followed by the immediate harvest of the hippocampus. We have previously shown that mice exposed to the EE for 2 h exhibited an increase of total and phosphorylated GluA1 at Ser831 and Ser845 in the mouse forebrain PSD fractions (49). Since the concept of EE was originally introduced by Donald Hebb (63), many studies have shown that EE has a considerable number of effects including gene expression, transcription, and translation, throughout the brain (64). Although further studies need to be conducted to answer why AMPARs, NMDARs, and phospho-GluA1 increased after short exposure to the training platform, our results suggest that appropriate cohorts that are exposed to the same behavioral apparatus without external stimulation, in this study the Walk group should be set as a control group for memory assessment and biochemical validation.

A central question for all forms of synaptic plasticity is the degree to which phenotypic changes are driven by changes in protein expression and/or PTMs, such as phosphorylation. A previous study indicates a requirement for protein synthesis and enhanced levels of protein phosphorylation for synaptic plasticity (3, 14, 65). However, the exact time frames that distinguish protein synthesis- or phosphorylation-dependency for learning and memory formation remains unclear (66, 67). In this study, we analyzed proteins from hippocampal PSD harvested 1 h after IA training or ∼5 min after immediate shock. Interestingly, One of the most interesting changes was a significant decrease in protein and phosphorylation levels followed by both IA training and immediate shock. Likewise, we observed a decreasing pattern of protein phosphorylation levels after IA training (Fig. 3*C*). The degree of decrease was more obvious for the phosphorylation level (>70% dephosphorylated) than the total protein level (approximately 25%). Methodologically, IA triggers sequential biochemical reactions in the hippocampus that are important for memory formation, and these biochemical events are similar to those necessary for synaptic plasticity including LTP (68). LTP is the most studied form of synaptic plasticity and it is the most closely linked molecular mechanism underlying learning and memory. LTP triggers various changes in the postsynaptic sites of neurons including gene expression, neuronal morphology, protein transportation, and ion channel properties. LTP in the hippocampus is a well-established model for learning and memory (69, 70). It was shown that LTP induced by learning *in vivo* mimicked the effects of hippocampal LTP induced by high-frequency stimulation (5, 71, 72). One of the key regulators of these neuronal processes occurring during LTP is protein phosphorylation (16, 73). However, our results show an overall trend of dephosphorylation in both IA and Shock groups. Because the degree of dephosphorylation is much bigger than reductions in protein level, increased protein phosphatase activity following IA training and immediate shock can be one of the possible mechanisms to explain our results. However, it should be noted that the molecular mechanisms of reduced phosphorylation levels in the PSD following IA or immediate shock need to be validated to uncover whether this is reflecting the effect of reduced protein level or other molecular events such as protein dephosphorylation. Among the protein components at the synapse, enzymes controlling protein phosphorylation have been considered important for the induction and maintenance of long-term changes in synaptic strength, and, as a counterpart, protein phosphatases have emerged as another key regulator of synaptic plasticity (14, 15). Especially, we highlight that the dephosphorylation of Ppm1h at S210 after cFC might be important for parallel signaling events between Hebbian and homeostatic changes to control the balance of the relative synaptic activity in fear-conditioned hippocampal PSD. While the functional role of Pppm1h remains uncertain, researchers recently reported that Ppm1h can counteract LRRK2 signaling *via* Rab protein dephosphorylation, which may potentially link to the molecular mechanisms of LRRK2-mediated neurological disorders such as Parkinson’s disease (74). Interestingly, the structure of Ppm1h has been predicted to be a docked model of a heterotetrameric complex through crosslinking and 3D docking (75). Ppm1h is a physiological dimer between a flexible loop 183 to 235 (75). This loop is situated near substrates at the active site of the dimeric partner (75). In our results, the phosphorylation of Ppm1h at S210 was significantly decreased after IA training. Thus, dephosphorylation of Ppm1h as S210 may lead to the activation of Ppm1h function, which is consistent with substantial experience-induced alteration corresponding to overall dephosphorylation patterns. Future studies will be needed to substantiate the function of how dimerization reflects in modulating Ppm1h activity in hippocampal neuron cells. Further, we demonstrated that Ppm1h can manipulate levels of glutamate receptors and is affected by neuronal activity both *in vivo* and *in vitro* (Fig. 7). The result on Ppm1h functionality is a good example supporting how global dephosphorylation of hippocampal PSD proteome affects IA-mediated learning and memory.

Interestingly, the phosphorylation of Ppm1h at S210 is most likely phosphorylated by CaMKI in neuron-like cells (76). We also showed that the PSD proteome underwent dynamic phosphorylation regulation following IA training and immediate shock and this led us to investigate kinases in the PSD (Fig. 6). More than 500 kinases are expressed in adult mammalian brains but only a few kinases, such as CaMKII, ERK1/2, PKA, PKG, PI3K, and Gsk3α/3β, are known to play critical role in learning and memory (73, 77). We demonstrated that 26 kinases and their phosphosites were dynamically regulated by IA training or immediate shock. We found that CaMKIIδ protein expression was increased in IA and further increased in the shock group. The roles of CaMKIIδ in the memory process remain unclear, but there is increasing evidence suggesting that this enzyme can be regulated by training and may contribute to different stages of memory formation. For example, it was found that sustained expression of CaMKIIδ was observed up to 1 week after novel object recognition training, and antisense oligo to a CaMKIIδ reversed the effect on memory persistence (78). In this training paradigm, transcriptional activation *via* NF-κB and increased histone acetylation in the promoter region of the *Camk2d* gene resulted in increase of CaMKIIδ expression beyond memory consolidation (79). Our results also support the hypothesis that the level of CaMKIIδ could be differentially regulated in different subcellular fractions following different types of behavioral task. We have also demonstrated that a series of kinases and their phosphosites were differentially regulated by IA training or immediate shock (Fig. 6). Functions and detailed molecular mechanisms of these kinases will need to be tested.

In summary, we believe that the dataset from our current study can be used broadly to study the underlying mechanisms for learning and memory formation. In summary, we applied a modified iTRAQ-based TiSH protocol to mouse hippocampal PSD fractions and provided a comprehensive phosphoproteomic dataset containing hundreds of proteins that showed changes in expression and/or site-specific phosphorylation following IA training or immediate shock. We observed a significant decrease in PSD proteome and phosphoproteome and the dynamic regulation of synaptic kinases and phosphatases. These results should be interpreted with some caution as we only analyzed PSD samples at the 1 h post-training time point. Therefore, we do not know how these findings generalize to other post-training time points, or whether these phenomena specifically represent the proteome remodeling during the early phase of memory formation. Therefore, in future studies, it will be interesting to monitor proteome and PTMome dynamics at multiple time scales to identify key modulators regulating memory formation and its maintenance. Taken together, we believe that the dataset from our current study can be used broadly to study the underlying mechanisms for learning and memory formation.

## Data Availability

The authors declare that all data supporting the findings of this study are available within the article. The proteomics data and search results associated with this study have been deposited to the ProteomeXchange Consortium (80) *via* the PRIDE (81) partner repository with the dataset identifier PXD045496, project DOI 10.6019/PXD045496 and project name “Experience-induced remodeling of the hippocampal post-synaptic proteome and phosphoproteome”. The reviewer account details are as follows; Username: reviewer_pxd045496@ebi.ac.uk, Password: MCFMYWxT.

## Supplemental data

This article contains supplemental data.

## Conflict of interest

R. L. H. is scientific cofounder and Scientific Advisory Board (SAB) member of Neumora Therapeutics and SAB member of MAZE Therapeutics. S. H., T. K., A. M. B., and M. R. L. declare no competing interests.
