# Supplementary material for: Experience-Induced Remodeling of the Hippocampal Post-synaptic Proteome and Phosphoproteome
Source: Mol Cell Proteomics. 2023 Oct 6;22(11):100661. doi: 10.1016/j.mcpro.2023.100661 (PMC10652125; doi:10.1016/j.mcpro.2023.100661)
Supplement: Supplemental Figure [file mmc5.pdf]

Supplementary Figure 1.

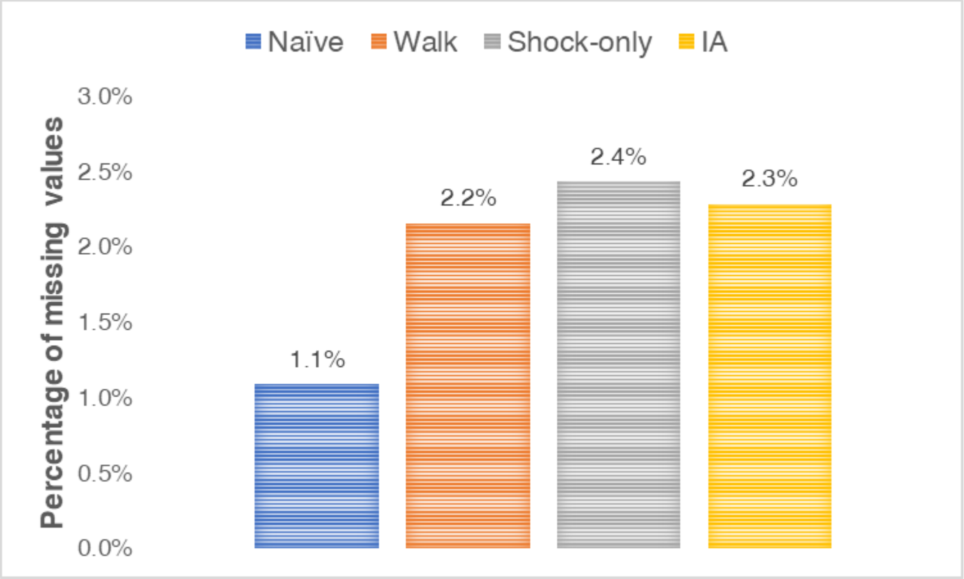

Supplementary Figure 1A, bar chart to show percentages of missing values for each PSD iTRAQ channel as low as ≤2.4% calculated at the PSM level.

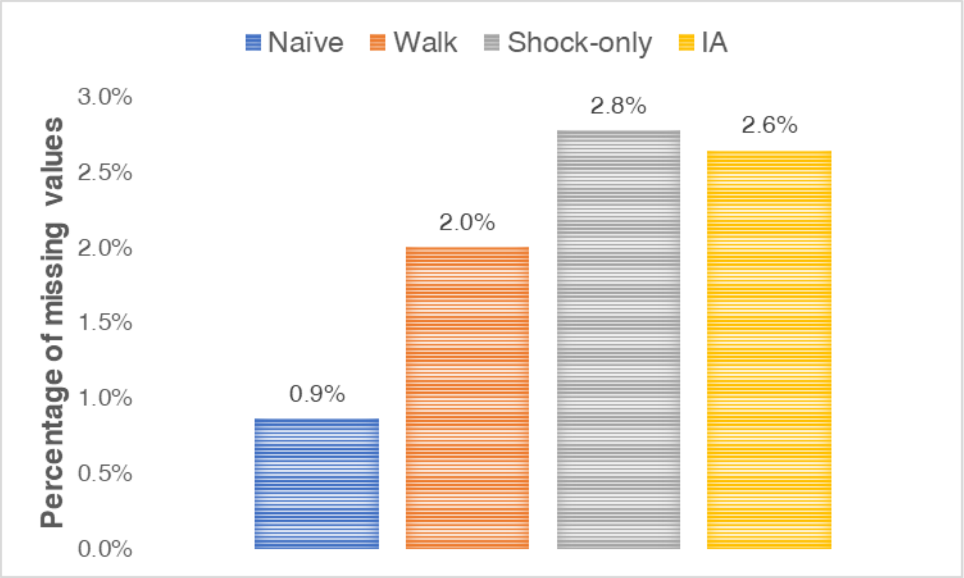

Supplementary Figure 1B, bar chart to show percentages of missing values for each PSD iTRAQ channel as low as ≤2.8% calculated at the phosphoPSM level.
